# TMC6 functions as a GPCR-like receptor to sense noxious heat via Gαq signaling

**DOI:** 10.1038/s41421-024-00678-9

**Published:** 2024-06-18

**Authors:** Chen Zhang, Fang Tong, Bin Zhou, Mingdong He, Shuai Liu, Xiaomeng Zhou, Qiang Ma, Tianyu Feng, Wan-Jie Du, Huan Yang, Hao Xu, Lei Xiao, Zhen-Zhong Xu, Cheng Zhu, Ruiqi Wu, Yan-Qing Wang, Qingjian Han

**Affiliations:** 1 Shanghai Stomatological Hospital & School of Stomatology, State Key Laboratory of Medical Neurobiology and MOE Frontiers Center for Brain Science, Institutes of Brain Science, Department of Integrative Medicine and Neurobiology, School of Basic Medical Science, Fudan University, Shanghai, China; 2 School of Brain Science and Brain Medicine, Zhejiang University School of Medicine, Hangzhou Zhejiang, China; 3 Tianjin Key Laboratory of Function and Application of Biological Macromolecular Structures, School of Life Sciences, Tianjin University, Tianjin, China; 4 Shanghai Key Laboratory of Craniomaxillofacial Development and Diseases, Fudan University, Shanghai, China

**Keywords:** Calcium signalling, Stress signalling

## Abstract

Thermosensation is vital for the survival, propagation, and adaption of all organisms, but its mechanism is not fully understood yet. Here, we find that TMC6, a membrane protein of unknown function, is highly expressed in dorsal root ganglion (DRG) neurons and functions as a Gαq-coupled G protein-coupled receptor (GPCR)-like receptor to sense noxious heat. TMC6-deficient mice display a substantial impairment in noxious heat sensation while maintaining normal perception of cold, warmth, touch, and mechanical pain. Further studies show that TMC6 interacts with Gαq via its intracellular C-terminal region spanning Ser^780^ to Pro^810^. Specifically disrupting such interaction using polypeptide in DRG neurons, genetically ablating Gαq, or pharmacologically blocking Gαq-coupled GPCR signaling can replicate the phenotype of TMC6 deficient mice regarding noxious heat sensation. Noxious heat stimulation triggers intracellular calcium release from the endoplasmic reticulum (ER) of TMC6- but not control vector-transfected HEK293T cell, which can be significantly inhibited by blocking PLC or IP3R. Consistently, noxious heat-induced intracellular Ca^2+^ release from ER and action potentials of DRG neurons largely reduced when ablating TMC6 or blocking Gαq/PLC/IP3R signaling pathway as well. In summary, our findings indicate that TMC6 can directly function as a Gαq-coupled GPCR-like receptor sensing noxious heat.

## Introduction

Organisms have developed dynamic and versatile mechanisms to detect various environmental stimuli. In most cases, a single modality of stimulus can be detected by multiple receptors with distinct properties and mechanisms. For example, light can be detected by ion channels (e.g., channelrhodopsins) in algae, pumps (e.g., halorhodopsin, bacteriorhodopsin) in bacteria, and G protein-coupled receptors (GPCRs) (e.g., retinal rhodopsin) in mammals^1,2^. Similarly, the mechanical force can be perceived by mechanosensitive ion channels (e.g., PIEZO1/2, OSCA/TMEM63)^3–5^ and GPCRs (e.g. GPR68)^6,7^. Thermosensation is vital for the survival, propagation, and adaption of all organisms, ranging from bacteria to mammals^8,9^. Significant progress has been made in identifying thermosensors in recent decades. Ion channels, such as TRP channels, Cl^–^ channel anoctamin 1 (ANO1), heat-activated stromal interaction molecule 1 (STIM1)-ORAI1, and two-pore-domain K^+^ (K2P) channels, have been identified as thermosensors that directly respond to thermal stimulation, resulting in inward/outward current or calcium influx in heterologous systems^8,10–12^. However, genetic ablation or silencing of these channels individually, in combination, or even in triple combinations only partially or mildly impairs thermosensation in mice^9,13–16^, suggesting the complexity of in vivo thermosensation mechanisms and the existence of alternative thermosensors. Recently, increasing evidence has shown the involvement of GPCRs and G-protein signaling in thermosensation as well. For instance, GLR-3/Gluk2 can sense temperatures lower than 18 °C through Gαi/o-signaling^17^. In *Drosophila*, rhodopsin (Rh1, Rh5, and Rh6), phospholipase Cβ (PLCβ), and downstream phosphoinositide metabolism enzymes are all involved in sensing innocuous warmth in the range of 18–24 °C^18,19^. However, studies investigating whether evolutionarily equivalent noxious heat-responding GPCRs exist in mammals are lacking. The TMC subfamily, consisting of TMC1–8, was initially identified 20 years ago through positional cloning of hereditary deafness genes and sequence homology searches^20,21^. While TMC1 and TMC2 have been established as mechanosensitive ion channels located at the tips of hair cell stereocilia, involved in auditory transduction^22–24^, the functions of the other TMCs have remained largely unknown. In this study, we have identified a novel sensor of noxious heat, TMC6, which functions as a GPCR-like receptor to sense noxious heat through the Gαq-PLC-IP3R-Ca^2+^ signaling pathway.

## Results

### *Tmc6*-deficient mice have defects in noxious heat sensation

**Fig. 1:**
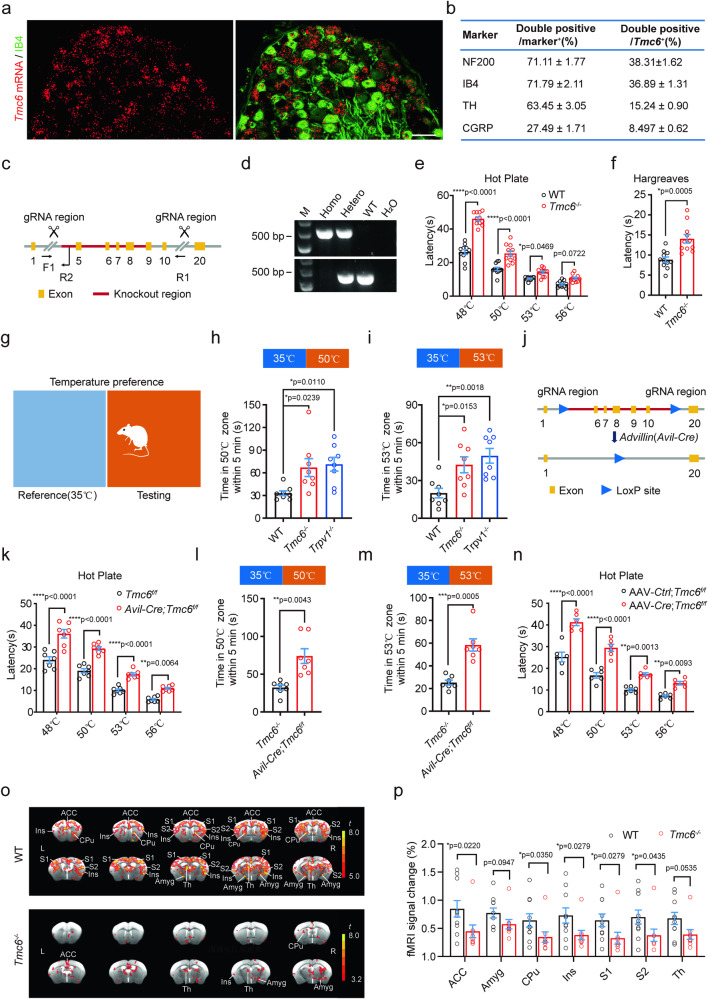
*Tmc6*-deficient mice have defects in thermosensation. **a**, **b** ISH and immunostaining tested the expression of *Tmc6* in mouse DRG. **a** ISH with probe targeting mouse *Tmc6* followed by immunostaining of nonpeptidergic DRG neuron marker FITC-conjugated IB4. Scale bar, 50 μm. **b** Cell type characterization of the expression of *Tmc6* in large- and medium-sized DRG neurons (NF200^+^), C-LTMRs (TH^+^), nonpeptidergic DRG neurons (IB4^+^), and peptidergic DRG neurons (CGRP^+^). *n* = 80 DRGs from 3 mice. **c** Schematic diagram of the strategy for generating *TMC6* global knockout (*Tmc6*^−/−^) mice. **d** Genotyping of *Tmc6*^−/−^ mice. Homozygotes: single band at 500 bp; Heterozygotes: two bands at 500 bp and 450 bp separately; WT allele: one band at 450 bp. **e**, **f** Behavioral test of WT mice and *Tmc6*^–/–^ mice. *n* = 11 mice/group. **e** Hot plate test at temperature 48 °C, 50 °C, 53 °C, and 56 °C. Two-way ANOVA followed by Sidak’s multiple comparisons test. **f** Hargreaves test with 35% irradiation intensity. Unpaired *t*-test. **g**–**i** Temperature preference test of WT, *Tmc6*^–/–^ and *Trpv1*^–/–^ mice. *n* = 8 mice/group. **g** Schematic diagram for the temperature preference test, the reference temperature (blue zone) was set at 35 °C, and the testing temperature (orange zone) was set at 50 °C (**h**) or 53 °C (**i**), the time mice spent in the testing zone within 5 min was counted. One-way ANOVA followed by Dunnett’s multiple comparisons test. **j** Schematic diagram of the strategy for generating *Avil-Cre;Tmc6*^*f/f*^ mice. **k**–**m** Behavioral test of *Avil-Cre;Tmc6*^*f/f*^ mice. *n* = 7 mice/group. **k** Hot plate test at temperature 48 °C, 50 °C, 53 °C, and 56 °C. Two-way ANOVA followed by Sidak’s multiple comparisons test analysis. Temperature preference test with a testing temperature of 50 °C (**l**) or 53 °C (**m**), the time mice spent in the testing area within 5 min was shown, Welch’s *t*-test. **n** Hot plate test of AAV-*Cre;Tmc6*^*f/f*^ mice at 48 °C, 50 °C, 53 °C, and 56 °C. Two-way ANOVA followed by Sidak’s multiple comparisons test analysis. *n* = 6 mice/group. **o**, **p** Functional magnetic resonance imaging (fMRI) test of WT and *Tmc6*^–/–^ mice. **o** The upper and lower set of images are the activation maps of WT mice brain and *Tmc6*^–/–^ mice brain, respectively, relevant brain regions were shown. L, left; R, right. Scale bar, *t* value range. **p** The comparison of signal changes between WT mice and *Tmc6*^–/–^ mice was performed. Mann–Whitney *U* test. *n* = 9–10 mice/group. All data are expressed as mean ± s.e.m. *p < 0.05, **p < 0.01, ***p < 0.001 and ****p < 0.0001.

To investigate the potential involvement of TMCs in somatosensation, we examined their expression in the dorsal root ganglion (DRG), the site where cell bodies of primary sensory neurons are located. Interestingly, we observed significantly higher expression of TMC6 in the DRG compared to other TMCs (Supplementary Fig. S1a). Additionally, the expression of TMC6 in DRG is markedly higher than in the spinal cord (SC), thalamus, and somatosensory cortex (Supplementary Fig. S1b). To further characterize the cell type specificity of TMC6 in the DRG, we performed in situ hybridization (ISH) combined with immunostaining, which revealed that TMC6 was predominantly expressed in DRG neurons without cell type specificity (Fig. 1a, b and Supplementary Fig. S1c, d).

To elucidate the role of TMC6 in somatosensation, we generated *Tmc6*^−/−^ mice through targeted deletion of a genomic region encoding amino acids 90^Gly^ to 408^Lys^, covering the first three presumptive transmembrane regions (Fig. 1c). This deletion not only removed 319 amino acids but also introduced a frameshift after the deleted segment (Fig. 1c). The successful targeting was confirmed by genotyping, RT-PCR with primers spanning 1570 to 1747 nt of full-length *Tmc6* mRNA, and ISH using a probe against full-length *Tmc6* mRNA spanning from 601 to 1501 nt (Fig. 1d and Supplementary Fig. S1e, f). *Tmc6*^−/−^ mice displayed normal overall appearance, viability, and overt behavior. Breeding between heterozygous animals resulted in wild-type (WT), heterozygous, and homozygous mutant offspring in expected Mendelian ratios. The proportions of calcitonin gene-related peptide-positive (CGRP^+^) (peptidergic DRG neurons), isolectin B4-positive (IB4^+^) (nonpeptidergic DRG neurons), neurofilament 200-positive (NF200^+^) (A-fiber generating larger and medium-sized DRG neurons), and tyrosine hydroxylase-positive (TH^+^) (C-fiber low threshold mechanoreceptors, C-LTMRs)^25–27^ immunoreactive neurons to the total number of DRG neurons were similar between WT and *Tmc6*^−/−^ mice (Supplementary Fig. S2a, b). Furthermore, the projection of CGRP^+^, IB4^+^, and NF200^+^ axonal terminals in the SC, as well as the skin innervation of protein gene product 9.5-positive (PGP9.5^+^) nerve fibers, were normal in *Tmc6*^−/−^ mice (Supplementary Fig. S2c–f). Additionally, no neuronal loss was observed in the SC (Supplementary Fig. S2g, h). Based on these findings, we conclude that TMC6 does not play a role in the development of DRG neurons and spinal neurons.

In comparison to littermate WT mice, *Tmc6*^−/−^ mice exhibited normal locomotion assessed by open field and rotarod test (Supplementary Fig. S3a–d), sensitivity to mechanical pain as assessed by graded Von Frey filaments (Supplementary Fig. S3e), touch sensitivity as assessed by the adhesive removal test (Supplementary Fig. S3f), evaporation cooling sensitivity as assessed by the acetone test (Supplementary Fig. S3g), and cold pain as assessed by the dry ice test (Supplementary Fig. S3h). Notably, *Tmc6*^−/−^ mice exhibited significantly impaired basal heat sensitivity in hot plate tests and Hargreaves tests, as indicated by increased withdrawal latency (Fig. 1e, f). In the temperature preference tests, mice were allowed to freely move between two zones with one held at 35 °C (Reference zone) and the other ranging from 5 °C to 56 °C (Testing zone) (Fig. 1g). The duration spent in the testing zone was recorded over 5 min, 10 min, and 15 min period separately. Results demonstrated that WT mice clearly avoided the testing zone when the temperature was set at 50 °C and 53 °C (Fig. 1h, i). In line with the aforementioned thermosensation tests, *Tmc6*^−/−^ mice exhibited significantly reduced avoidance from the testing zone, similar to the phenotype observed in *Trpv1*^−/−^ mice (Fig. 1h, i and Supplementary Fig. S3i–l). However, the avoidances of *Tmc6*^−/−^ mice in response to warmth (41 °C) and cold (5 °C, 15 °C) were comparable to those of WT mice (Supplementary Fig. S3m–r). To ascertain the specific involvement of TMC6 in DRG neurons contributing to the impairment of noxious heat sensation, we generated *Advillin*-*Cre;Tmc6*^*f/f*^ (*Avil-Cre;Tmc6*^*f/f*^*)* mice (Fig. 1j and Supplementary Fig. S4a), which selectively lack TMC6 in primary sensory neurons, encompassing DRG neurons and trigeminal ganglion (TG) neurons. Behavioral tests, including the hot plate test, Hargreaves test, temperature preference test, dry ice test, acetone test, Von Frey test, and adhesive removal test, demonstrated that noxious heat sensation, but not innocuous cold, cool, warmth, and mechanical sensation, was affected in the *Avil-Cre;Tmc6*^*f/f*^ mice, aligning with the observations in *Tmc6*^−/−^ mice (Fig. 1k–m and Supplementary Fig. S4b–j). These findings strongly suggest that TMC6 expressed in DRG neurons, rather than in other tissues or cell types, mediates noxious heat sensation.

To eliminate potential compensatory effects arising from embryonic TMC6 ablation, we adopted a strategy involving the transient deletion of TMC6 in DRG neurons. This was achieved by utilizing P0 *Tmc6*^*f/f*^ mice and administering intraperitoneal injections of AAV2/9-*Cre* at a titer of 5 × 10^12^ vg/mL. Notably, this method demonstrated high specificity for infecting ganglion neurons, including those in the DRG and TG, while inducing minimal impact on neurons within the SC or brain (Supplementary Fig. S5). The latency of hot plate test and Hargreaves test significantly increased after transiently ablating TMC6 in DRG neurons but did not affect the sensation of cold, cool, and mechanical sensation (Fig. 1n and Supplementary Fig. S6), which is consistent with that of *Tmc6*^−/−^ mice and *Avil-Cre;Tmc6*^*f/f*^ mice, supporting the conclusion that TMC6 expressed in DRG neurons mediates noxious heat sensation.

Noxious heat stimulation can induce robust brain activity in various brain regions, including the primary (S1) and secondary (S2) somatosensory cortex, insular (Ins) cortex, amygdala (Amyg), and striatum (caudate nucleus and putamen, CPu)^28^. To investigate whether the sensation to noxious heat was altered in *Tmc6*^−/−^ mice, we conducted functional magnetic resonance imaging (fMRI). The results revealed that in WT mice, noxious heat stimuli (48 °C) applied to the left hind paw triggered strong and widespread activation in multiple brain regions, including bilateral S1, S2, insula, anterior cingulate cortex (ACC), CPu, and thalamus (Th). In contrast, the response to the same stimulation in *Tmc6*^−/−^ mice was significantly reduced, with only weak activations observed in the ACC, contralateral insula, and contralateral Amyg (Fig. 1o). To quantify the specific differences between the WT mice and *Tmc6*^−/−^ mice in these nociceptive brain regions, we statistically compared the signal changes of each area. The activation magnitude in the WT group was nearly twice that of the *Tmc6*^−/−^ group. The magnitude of activation in S1 (*p* = 0.028), S2 (*p* = 0.043), ACC (*p* = 0.022), Ins (*p* = 0.028), and CPu (*p* = 0.035) in response to the noxious heat stimulation was significantly lower in *Tmc6*^−/−^ mice compared to WT mice. However, although the activations were greater in WT mice than in *Tmc6*^−/−^ mice, the differences in the Amyg and Th were not statistically significant (Fig. 1p). Taken together, these findings suggest that *Tmc6*^−/−^ mice exhibit impairments in noxious heat sensation, as evidenced by attenuated brain responses to noxious heat stimuli.

### *Tmc6* deficiency in DRG neurons impairs noxious heat-elicited Ca^2+^ response and neuronal firing

**Fig. 2:**
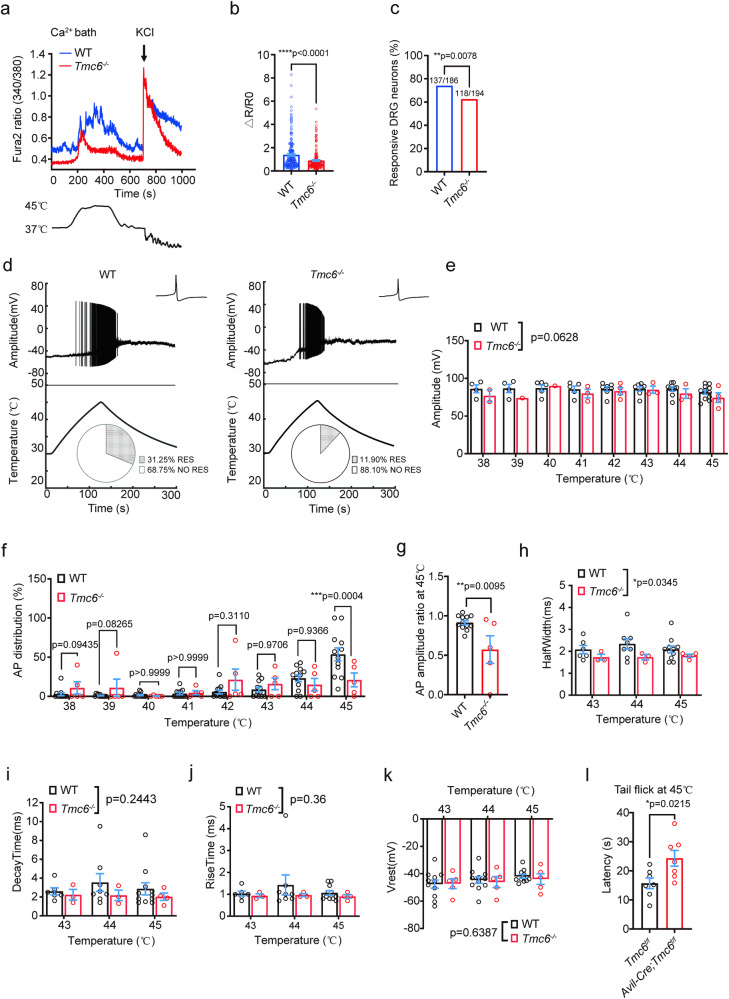
*Tmc6*^−/−^ DRG neurons have defects in responding to noxious heat stimulation. **a**–**c** Fura-2 ratiometric calcium imaging was performed on WT and *Tmc6*^−/−^ DRG neurons, heating from 37 to 45 °C. **a** Representative traces illustrating heat-induced Fura2 ratio (340/380) in cultured DRG neurons from WT mice and *Tmc6*^−/−^ mice. **b** Statistical analysis of heat-induced Fura2 ratio (340/380) changes ($\Delta$R/R0) were presented as bar graphs combined with scatter dot plots. Mann–Whitney *U* test. **c** The responsive percentage of WT and *Tmc6*^−/−^ DRG neurons to heating, where positive response was defined as $\Delta$R/R0 > 5 times of s.e.m during heating. *χ*^2^ test, *n* = 186–194 neurons/group, with 9–11 DRG neuron preparations. **d**–**k** Heat-evoked AP firing was recorded in small DRG neurons with the bath solution temperature increased from 30 to 45 °C. **d** Representative traces of heat-evoked APs in small-sized DRG neurons of WT and *Tmc6*^−/−^ mice. The percentage of noxious heat-responsive DRG neurons in WT (31.25%, *n* = 48 neurons) and *Tmc6*^−/−^ DRG neurons (11.90%, *n* = 42), *χ*^2^ test, *p* = 0.0276. **e** Amplitudes of AP during heating from 38 to 45 °C. Two-way ANOVA. *n* = 5–12 neurons/group. **f** Distribution of AP firing was shown as the percentage of spikes at each degree from 38 to 45 °C. Two-way ANOVA followed by Sidak’s multiple comparisons test. *n* = 5–12 neurons/group. **g** The ratio of AP amplitude at 45 °C to the first AP. Unpaired *t*-test. *n* = 5–12 neurons/group. **h**–**k** AP kinetics of WT and *Tmc6*^−/−^ DRG neurons at each degree from 43 to 45 °C were calculated and plotted: AP half width (**h**), AP decay time (**i**), AP rise time (**j**), and the basal membrane potential (**k**). Two-way ANOVA. *n* = 5–12 neurons/group. **l** Tail-flick test of *Avil-Cre;Tmc6*^*f/f*^ mice at 45 °C. Unpaired *t*-test. *n* = 7 mice/group. All data are presented as mean ± s.e.m. *p < 0.05, **p < 0.01, ***p < 0.001 and ****p < 0.0001.

We tested noxious heat-induced Ca^2+^ response on cultured DRG neurons. In a bath solution containing Ca^2+^, noxious heat induced a robust Ca^2+^ response in cultured WT DRG neurons, and the change of Fura2 ratio (ΔR/R0) in recorded *Tmc6*^−/−^ DRG neurons exhibited a noticeable decrease (Fig. 2a, b). Furthermore, the percentage of responsive DRG neurons significantly decreased in the *Tmc6*^−/−^ group (Fig. 2c; 137/186 WT DRG neurons vs 118/194 *Tmc6*^−/−^ DRG neurons. *χ*^2^ test, *p* = 0.0078).

In our investigation of stepped heat-evoked action potential (AP) discharges in DRG neurons, we subjected small neurons (10–20 μm in diameter) from both WT and *Tmc6*^−/−^ mice to heat ramps ranging from 30 °C to noxious levels at 45 °C. Strikingly, the *Tmc6*^−/−^ group exhibited a dramatic reduction in the percentage of noxious heat-sensitive DRG neurons (31.25% for WT DRG neurons, *n* = 48; 11.90% for *Tmc6*^*−/−*^ DRG neurons, *n* = 42; *χ*^2^ test, *p* = 0.0276) (Fig. 2d). Analysis of the AP amplitudes from 38 °C to 45 °C revealed a significant decreasing trend of AP amplitude in *Tmc6*^−/−^ DRG neurons with a *p* value of 0.0628 (Fig. 2e). Subsequently, we examined the distribution pattern of APs by assessing the percentage of AP numbers at each degree. Notably, the AP firing rate in small DRG neurons of *Tmc6*^−/−^ mice exhibited a remarkable decrease at 45 °C (Fig. 2f). When normalized to the amplitude of the first AP, the normalized AP amplitude at 45 °C significantly decreased from 0.9136 ± 0.0258 in WT DRG neurons (*n* = 48) to 0.5717 ± 0.1750 in *Tmc6*^−/−^ DRG neurons (*n* = 42) (Fig. 2g). Additionally, we performed kinetic analysis of single AP. Due to the fewer number of APs induced at temperatures below 43 °C, we only present the statistical results from 43 °C to 45 °C. Compared to the control group, the half-height-duration of APs in *Tmc6*^−/−^ DRG neurons was significantly reduced (Fig. 2h). Although there were no significant differences in rise time and decay time, the decay time in *Tmc6*^−/−^ DRG neurons showed a noticeable decreasing trend compared to the control group (Fig. 2i, j). We also analyzed the membrane potentials of the APs. The membrane potentials of both groups decreased as the temperature increased, but there was no significant difference between the WT group and *Tmc6*^−/−^ group (Fig. 2k). This indicates that *Tmc6*^−/−^ small DRG neurons display attenuated AP firing during noxious heat stimulation. Consistent with these cellular findings, the latency of the tail-flick test at 45 °C was significantly increased in *Avil-Cre;Tmc6*^*f/f*^ mice (Fig. 2l). This alignment between cellular responses and behavioral outcomes further supports the role of TMC6 in the sensitivity of DRG neurons to noxious heat stimuli.

To investigate how TMC6 mediated noxious heat-elicited Ca^2+^ response and neuronal firing, we conducted calcium imaging on cultured DRG neurons to test whether TMC6 affects well-recognized noxious heat sensors including TRPV1, TRPA1, and TRPM3^14^. The results showed that specific agonists of TRPV1 (Capsaicin, 1 μM)-, TRPA1 (AITC, 200 μM)-, and TRPM3 (CIM0216, 1 μM)-elicited calcium responses were all not affected in *Tmc6*^−/−^ DRG neurons (Supplementary Fig. S7a–f). Additionally, the whole-cell sodium current of DRG neurons was also comparable between WT DRG neurons and *Tmc6*^−/−^ DRG neurons (Supplementary Fig. S7g, h). These data suggested that TMC6 mediated noxious heat-elicited Ca^2+^ response and AP firing, but didn’t affect TRPV1, TRPA1, TRPM3, or sodium channels.

### TMC6-mediated Ca^2+^ release from the endoplasmic reticulum (ER) contributes to AP generation elicited by noxious heat

**Fig. 3:**
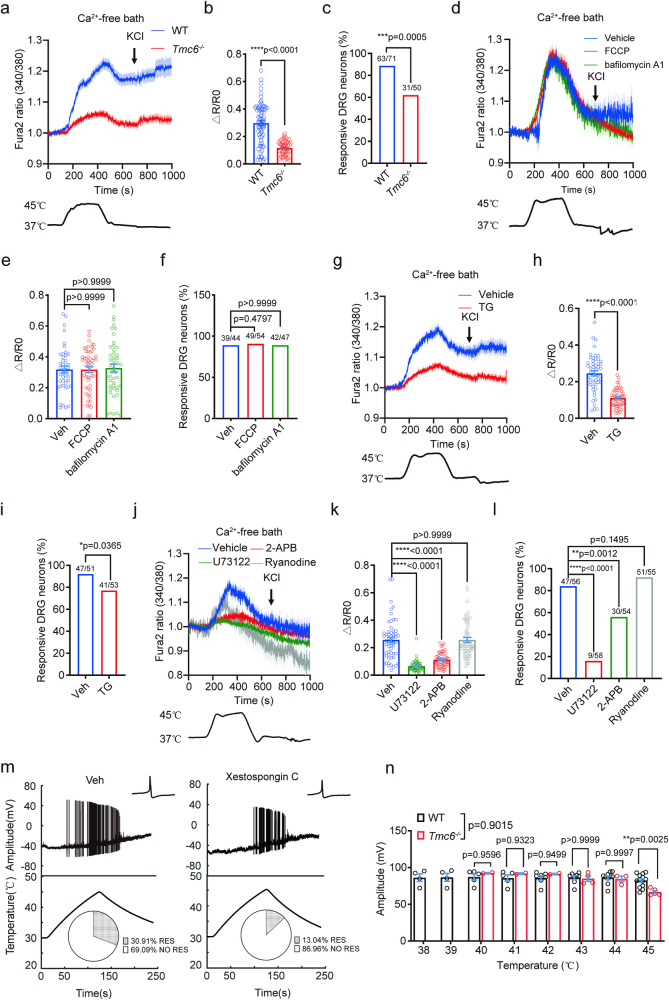
TMC6 mediates noxious heat-induced ER calcium release. **a**–**c** Fura-2 ratiometric calcium imaging of cultured DRG neurons from WT or *Tmc6*^−/−^ mice in Ca^2+^-free bath solution in response to heating from 37 to 45 °C. **a** Average traces of normalized Fura-2 ratios in WT and *Tmc6*^−/−^ DRG neurons. **b** Statistics of heat-induced $\Delta$R/R0, showing as bar graphs combined with scatter dot plots. Welch’s *t*-test. **c** The responsive percentage of WT and *Tmc6*^–/–^ DRG neurons to heating in Ca^2+^-free bath. *χ*^2^ test. *n* = 50–71 neurons, from 3 DRG neuron preparations. **d**–**f** Fura-2 ratiometric calcium imaging of cultured DRG neurons preincubated with FCCP or bafilomycin A1 in Ca^2+^-free bath solution in response to heating from 37 to 45 °C. **d** Average traces of normalized Fura-2 ratios (340/380) of neurons during heating, with separate preincubation using 3 μM FCCP for 5 min and 100 nM bafilomycin A1 for 15 min. **e** Statistics of heat-induced $\Delta$R/R0, showing as bar graphs combined with scatter dot plots. Kruskal–Wallis test followed by Dunn’s multiple comparison test analysis. **f** The responsive percentage of DRG neurons to heating in Ca^2+^-free bath. *χ*^2^ test. *n* = 44–54 neurons from 3 DRG neuron preparations. **g**–**i** Fura-2 ratiometric calcium imaging was conducted on cultured DRG neurons preincubated with TG in a Ca^2+^-free bath solution, in response to heating from 37 to 45 °C. **g** Average traces of normalized Fura-2 ratios (340/380) of neurons during heating, following preincubation with 2.5 µM TG for 15 min. **h** Statistical analysis of heat-induced $\Delta$R/R0. Welch’s *t*-test. **i** The responsive percentage of DRG neurons to heating in a Ca^2+^-free bath. *χ*^2^ test. *n* = 51–53 neurons from 3 DRG neuron preparations. **j**–**l** Fura-2 ratiometric calcium imaging of cultured DRG neurons preincubated with U73122, 2-APB, and Ryanodine in a Ca^2+^-free bath solution in response to heating from 37 to 45 °C. **j** Average traces of normalized Fura-2 ratios (340/380) of neurons during heating, with DRG neurons preincubated separately with 10 μM U73122 for 10 min, 20 μM 2-APB for 10 min, or 10 μM Ryanodine for 20 min. **k** Statistical analysis of heat-induced $\Delta$R/R0. Kruskal–Wallis test followed by Dunn’s multiple comparisons test analysis. **l** The responsive percentage of DRG neurons to heating in Ca^2+^-free bath. *χ*^2^ test. *n* = 54–58 neurons from 3 DRG neuron preparations. **m**, **n** Heat-evoked AP firing was recorded in small DRG neurons with the bath solution temperature increased from 30 to 45 °C. **m** Representative traces of heat-evoked APs in small-sized DRG neurons of Veh group and Xestospongin C-treated group. The percentage of noxious heat-responsive DRG neurons in the Veh group (30.91%, *n* = 55) and Xestospongin C-treated group (13.04%, *n* = 46). *χ*^2^ test. *p* = 0.0276. **n** Amplitudes of AP during heating from 38 to 45 °C. Two-way ANOVA. *n* = 5–14 neurons/group. All data are expressed as mean ± s.e.m. *p < 0.05, **p < 0.01, ***p < 0.001 and ****p < 0.0001.

To further study the mechanism by which TMC6 mediates thermosensation, we tested noxious heat-elicited Ca^2+^ response in the absence of extracellular Ca^2+^. Strikingly, Ca^2+^ response can still be induced by noxious heat in a Ca^2+^-free bath solution indicating the intracellular Ca^2+^ release, which was significantly reduced in *Tmc6*^−/−^ DRG neurons (Fig. 3a, b). Additionally, there was a decrease in the proportion of responsive neurons (Fig. 3c). Consistently, overexpressing TMC6 in *Tmc6*^−/−^ DRG neurons by electroporation could rescue the reduction of noxious heat-elicited Ca^2+^ response in Ca^2+^-free bath solution (Supplementary Fig. S8a–c), suggesting the specific role of TMC6 in mediating Ca^2+^ release from internal stores.

Ca^2+^ serves as a crucial second messenger and is typically stored in intracellular organelles such as the mitochondrion, lysosome, and ER^29–35^. We then employed carbonyl cyanide-ρ-(trifluoromethoxy)-phenylhydrazone (FCCP; 3 µM) and bafilomycin A1 (BFA1; 100 nM) to selectively deplete calcium from the mitochondria and lysosomes, respectively^31,36,37^. Neither of these treatments affected the noxious heat-induced Ca^2+^ release from internal stores in the WT DRG neurons, as well as the proportion of responsive neurons (Fig. 3d–f). ER represents the primary intracellular calcium storage site responsible for calcium mobilization triggered by receptor signaling^34^. Notably, abolishing Ca^2+^ release from the ER using thapsigargin (TG; 2.5 µM) significantly reduced the noxious heat-induced Ca^2+^ response and the percentage of responsive neurons in cultured WT DRG neurons (Fig. 3g–i), suggesting that the ER store is the main source of noxious heat-elicited internal Ca^2+^ release. Considering the pivotal role of ryanodine receptors (RyRs) and Gαq signaling component inositol 1,4,5-trisphosphate receptors (IP3Rs) in ER calcium release, we examined the effects of cell-permeable inhibitors of RyRs (ryanodine; 10 µM) and IP3Rs (2-aminoethoxydiphenylborate or 2-APB; 20 µM) on heat-induced internal calcium release. Pre-treatment with 10 μM ryanodine did not affect the calcium responses elicited by heat, whereas 2-APB nearly abolished the calcium response in cultured WT DRG neurons under calcium-free conditions, in line with the percentage of responsive neurons (Fig. 3j–l). Consistently, specific blockade of another Gαq signaling component-phospholipase C (PLC) with U73122 effectively diminished noxious heat-induced intracellular Ca^2+^ release and the percentage of responsive neurons either (Fig. 3j–l), indicating Gαq signaling involved in noxious heat-induced Ca^2+^ release from ER.

To test whether the Ca^2+^ release from ER contributes meaningfully to heat sensation, we recorded stepped heat-evoked AP discharges in DRG neurons by applying heat ramps from 30 °C to noxious heat levels of 45 °C to small DRG neurons (10–20 μm in diameter) after blocking IP3R with its specific antagonist Xestospongin C (5 μM). Although the distribution of noxious heat-induced APs at each degree and the normalized amplitude of noxious heat-induced APs at 45 °C were not affected (Supplementary Fig. S8d, e), the percentage of noxious heat-sensitive DRG neurons (30.91% for Veh group, *n* = 55; 13.04% for Xestospongin C group, *n* = 46; *χ*^2^ test, *p* = 0.0330) (Fig. 3m) and the AP amplitudes elicited by heating from 38 °C to 45 °C dramatically reduced in Xestospongin C group compared with that of Veh group at 45 °C (Fig. 3n). These data showed that TMC6-mediated Ca^2+^ release from the ER contributes to the generation of noxious heat-elicited APs.

### TMC6 localizes on the cell membrane and mediates noxious heat-induced ER calcium release via Gαq-PLC-IP3R signaling

**Fig. 4:**
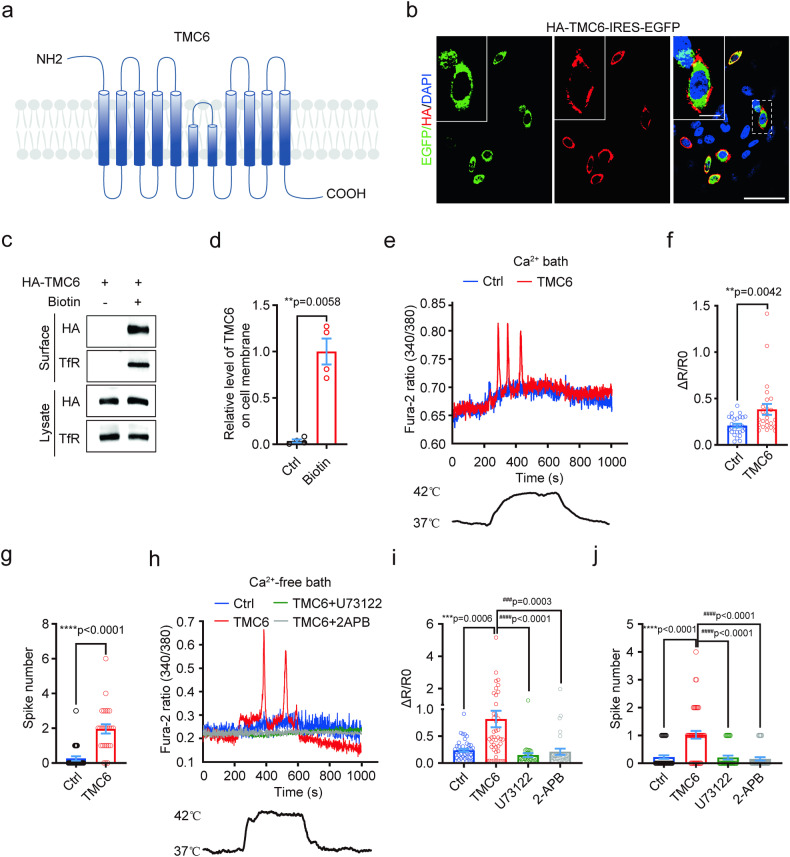
TMC6 localizes on the cell membrane and mediates heat stimulation-induced internal calcium release via Gαq signaling. **a** Schematic diagram illustrates the topology of TMC6. **b** Nonpermeable live cell labeling on CHO cells overexpressing N-terminal HA-tagged and C-terminal EGFP-tagged TMC6 with antibody against HA. Scale bars, 50 μm and 10 μm separately. **c**, **d** Cell surface biotinylation on CHO cells overexpressing HA-TMC6 and immunoblotted with antibodies against HA and transferrin receptor (TfR). **d** Quantitative analysis of surface biotinylation assay in **c**. Welch’s *t*-test. *n* = 4. **e**–**g** Fura-2 ratiometric calcium imaging of HEK293T cells overexpressing mouse TMC6 in response to heating from 37 to 42 °C. **e** Representative traces of heat-induced Fura2 ratio changes in HEK293T cells transfected with control vector or mouse TMC6. **f** Statistics of heat-induced $\Delta$R/R0. **g** Statistics of spike number during heating. Mann–Whitney *U* test. *n* = 25–27 cells from 3 transfections/group. **h**–**j** Fura-2 ratiometric calcium imaging of HEK293T cells overexpressing mouse TMC6 in Ca^2+^-free bath solution in response to heating from 37 to 42 °C. **h** Representative traces of heat-induced $\Delta$R/R0 in HEK293T cells transfected with control vector or mouse TMC6, or preincubated with 10 μM U73122 for 15 min, 20 μM 2-APB for 10 min. **i** Statistics of heat-induced $\Delta$R/R0. **j** Statistics of spike number during heating. Kruskal–Wallis test followed by Dunn’s multiple comparisons test. *n* = 34–45 cells from 3 transfections/group. All data are expressed as mean ± s.e.m. **p < 0.01, ***p < 0.001, ****p < 0.0001, ^###^*p* < 0.001 and ^####^*p* < 0.0001.

TMC6 is composed of a large extracellular N-terminal domain (NTD, Met^1^–Arg^203^) containing disordered regions, followed by ten transmembrane helices (TM1–TM10) and an intracellular C-terminal domain (CTD, Gly^740^–Pro^810^), as predicted by AlphaFold2, TMHMM, and OPM2 models (Fig. 4a and Supplementary Fig. S9a). Notably, TM8 exhibits a 180-degree bend across the lipid bilayer, resulting in the NTD and CTD of TMC6 being situated on opposite sides of the biomembrane (Fig. 4a and Supplementary Fig. S9a). Experimental evidence supports the localization of TMC6 on the cell membrane, with the N-terminal of TMC6 found in the extracellular space as confirmed by nonpermeable live cell labeling using antibodies against an N-terminal HA-tag (Fig. 4b). Moreover, both cell surface biotinylation assay and direct overexpression of EGFP-fused TMC6 validate the cell membrane localization of TMC6 (Fig. 4c, d and Supplementary Fig. S9b).

To investigate whether TMC6 regulated noxious heat-elicited Ca^2+^ release from ER via Gαq signaling, we conducted Ca^2+^ imaging on heterologous HEK293T cells. RT-PCR analysis revealed the absence of endogenous TMC6 expression in HEK293T cells, whereas human DRG cells exhibited detectable TMC6 expression (Supplementary Fig. S9c). Upon noxious heat stimulation, where the temperature increased from 37 to 42 °C, both TMC6-transfected and non-transfected cells exhibited a mild increase in intracellular calcium concentration ([Ca^2+^]i) (Fig. 4e). Interestingly, TMC6-transfected cells displayed a distinct cytoplasmic calcium spike response in addition to the background increase in [Ca^2+^]i, while non-transfected cells did not exhibit this spike response (Fig. 4e–g). These results indicate that TMC6 mediates the observed calcium spike response. To determine whether the TMC6-mediated cytoplasmic calcium spikes were a result of extracellular calcium influx or internal store release, we performed heat stimulation experiments under extracellular calcium-free conditions. Surprisingly, in both TMC6-transfected and non-transfected cells, the mild increase in [Ca^2+^]i disappeared when extracellular Ca^2+^ was absent. However, in TMC6-transfected cells, noxious heat still induced cytoplasmic Ca^2+^ spikes, and Ca^2+^ spikes were effectively blocked by an inhibitor of IP3R and PLC (Fig. 4h–j) indicating the necessity of Gαq signaling in TMC6-mediated noxious heat-elicited Ca^2+^ release from ER. Collectively, these data suggested that TMC6 mediated heat-induced ER Ca^2+^ release via Gαq signaling.

### TMC6 interacts with Gαq and mediates noxious heat-induced intracellular Ca^2+^ release

**Fig. 5:**
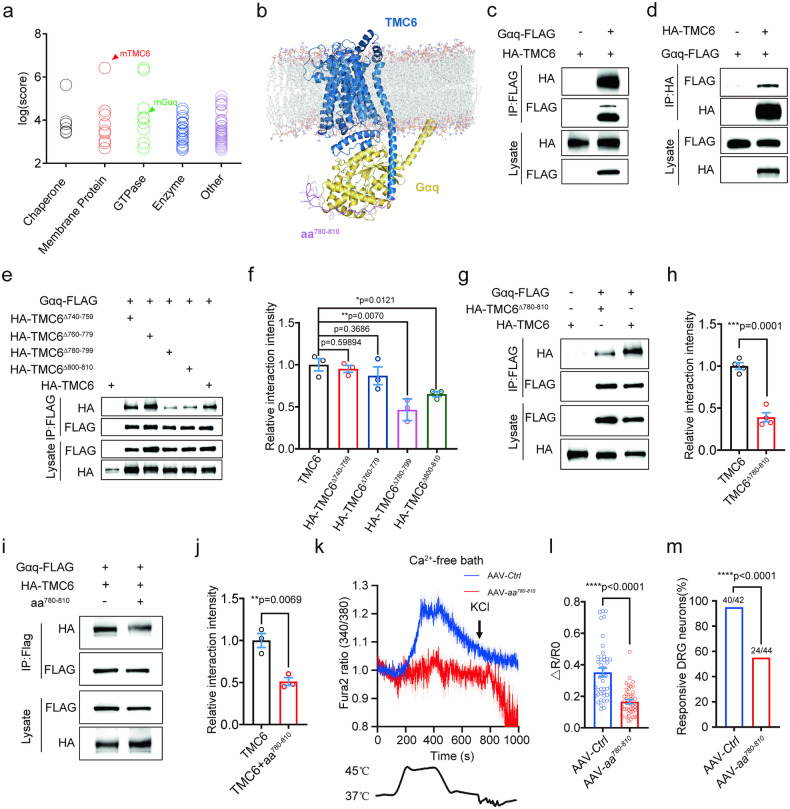
TMC6 interacting with Gαq and TMC6/Gαq signaling mediates noxious heat-induced Ca^2+^ release from internal store. **a** MS analysis of proteins co-precipitated with antibody against HA in the lysate of ND7/23 cells which overexpressed HA-tagged TMC6. **b** Computer simulation of the interaction between TMC6 and Gαq. **c**, **d** Lysate of ND7/23 cells overexpressing HA-tagged TMC6 and FLAG-tagged Gαq was immunoprecipitated with antibody against FLAG (**c**) or HA (**d**), and immunoblotted with antibody against HA or FLAG respectively. **e** Immunoprecipitation with antibody against FLAG in lysate of ND7/23 cells overexpressing Gαq-FLAG together with HA-TMC6, HA-TMC6^∆740–759^, HA-TMC6^∆760–779^, HA-TMC6^∆780–799^, or HA-TMC6^∆800–810^ separately. **f** Quantification of the interaction between Gαq-FLAG and HA-TMC6, or HA-TMC6 mutants. Unpaired *t*-test. *n* = 3. **g** Immunoprecipitation with antibody against FLAG in the lysate of ND7/23 cells coexpressing Gαq-FLAG together with HA-TMC6 or HA-TMC6^∆780–810^. **h** Quantification of the interaction between Gαq-FLAG and HA-TMC6, or HA-TMC6^∆780–810^. Unpaired *t*-test. *n* = 4. **i**, **j** Immunoprecipitation with antibody against FLAG in the lysate of ND7/23 cells overexpressing Gαq-FLAG together with HA-TMC6 and blocking peptide amino acids 780–810 in ND7/23 cells (**i**), and quantification of the interaction between HA-TMC6 and Gαq-FLAG (**j**). Unpaired *t*-test. *n* = 3. **k**–**m** Fura-2 ratiometric calcium imaging of cultured DRG neurons from mice intraperitoneally injected with AAV2/9-*aa*^*780*–*810*^ at P0 in Ca^2+^-free bath solution and stimulated with heating from 37 to 45 °C. **k** Average traces of normalized Fura-2 ratios showing heat-induced Fura2 ratio changes. **l** Statistics of heat-induced $\Delta$R/R0. Mann–Whitney *U* test. **m** The responsive percentage of DRG neurons to heating in Ca^2+^-free bath. *χ*^2^ test. *n* = 42–44 neurons from 3 DRG neuron preparations. All data are expressed as mean ± s.e.m. *p < 0.05, **p < 0.01, ***p < 0.001, and ****p < 0.0001.

To investigate how TMC6 mediate Gαq signaling, we conducted immunoprecipitation-mass spectrometry (IP-MS) to screen for binding partners of TMC6 in ND7/23 cells, a hybridized cell line consisting of mouse neuroblastoma and rat DRG neurons. We achieved this by overexpressing 2HA-TMC6 in ND7/23 cells (Supplementary Fig. S10a). Through IP-MS analysis, we identified a total of 115 differentially bound proteins in the HA antibody group, including common chaperones, membrane proteins, enzymes, and cytoskeletal proteins (Fig. 5a and Supplementary Table S1). Notably, we observed a high-confidence interaction between TMC6 and several G-proteins, particularly Gαq (Fig. 5a). To delineate the specific interaction regions between TMC6 and Gαq, we utilized computational modeling to obtain a complex structure using the AlphaFold multimer approach. Among the intracellular loops (ICL1–5) and the CTD, we identified the Ser^780^–Pro^810^ regions as anchoring interfaces for Gαq (Fig. 5b). Consequently, we generated four TMC6 truncations at the binding interface, deleting amino acids 740–759 (TMC6^∆740–759^), 760–779 (TMC6^∆760–779^), 780–799 (TMC6^∆780–799^), or 800–810 (TMC6^∆800–810^) of TMC6, respectively (Supplementary Fig. S10b). Then, we performed co-immunoprecipitation (Co-IP) assays to examine the interactions between FLAG-tagged Gαq and HA-tagged TMC6 or TMC6 truncations in the ND7/23 heterologous system. The bindings were readily detected using direct IP of Gαq with a primary antibody against FLAG and reverse IP of TMC6 with an antibody against HA (Fig. 5c, d). Furthermore, the Co-IP assay confirmed that the amino acids 780–810 region within the C-terminal of TMC6 is crucial for its interaction with Gαq, as the interaction between TMC6 and Gαq decreased by over 50% after the deletion of amino acids 780–810 (TMC6^∆780–810^) (Fig. 5e–h). Moreover, overexpression of the polypeptide amino acids 780–810 effectively competes with the interaction between full-length TMC6 and Gαq (Fig. 5i, j). These findings suggest that TMC6 interacts with Gαq through the C-terminal helix, spanning amino acids Ser^780^ to Pro^810^. Consistently, disruption of the interaction between TMC6 and Gαq by overexpressing the polypeptide amino acids 780–810 in DRG effectively diminished the heat-induced release of calcium from internal stores and the percentage of responsive neurons (Fig. 5k–m).

To gain insights into the dynamics of TMC6–Gαq interactions, we performed all-atom simulations of membrane-embedded TMC6 and its association with Gαq at the cytosolic side. Under regular culture temperature (303.15 K = 30 °C), Gαq exhibited stable association with the CTD of TMC6 throughout the 100 ns trajectory, with minimal distance fluctuations of less than 1 Å (red line, Supplementary Fig. S10c). However, at an elevated temperature of 323.15 K (50 °C), the average distance between TMC6 and Gαq increased, accompanied by larger fluctuations (black shaded area, Supplementary Fig. S10c), suggesting the disassociation of Gαq. Notably, we observed deformation of the Gβγ handle at the N-terminal of the Gαq subunit (Supplementary Fig. S10d). Based on these observations, we hypothesize that heat signals likely deform TMC6 and promote the release of Gαq for downstream signaling, resembling the kinetic activation of Gαq-coupled GPCRs^38^.

Collectively, these findings indicate that TMC6 directly interacts with Gαq and mediates intracellular Ca^2+^ release in Gαq signaling dependent manner.

### TMC6-mediated Gαq signaling is critical for noxious heat sensation

Next, we investigate the role of TMC6-mediated Gαq-PLC-IP3R-Ca^2+^ signaling in noxious heat sensation. Firstly, we tested the colocalization of TMC6, Gαq, and IP3R in DRG using ISH combined with immunohistochemistry (IHC). Our findings revealed that Gαq was mainly expressed in small diameter DRG neurons (Supplementary Fig. S11a). Subsequent cell-type profiling demonstrated that among Gαq^+^ DRG neurons, 4.10% were NF200^+^, 20.08% were CGRP^+^, 31.66% were IB4^+^, and 6.23% were TH^+^ (Supplementary Fig. S11b). These results indicate that Gαq expression is predominantly observed in small diameter DRG neurons. Through double ISH using probes against IP3R1 and Gαq, we observed that IP3R1 was generally expressed in DRG neurons, with most Gαq^+^ DRG neurons co-expressing IP3R1 (Supplementary Fig. S12).

**Fig. 6:**
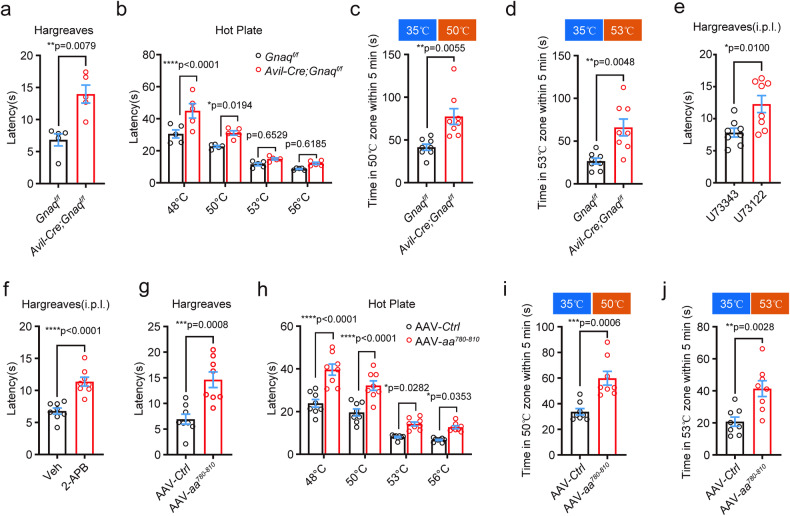
TMC6 mediates noxious heat sensation via Gαq signaling. **a** Hargreaves test with 35% irradiation intensity of *Avil-Cre;Gnaq*^*f/ff*^ mice. Mann–Whitney *U* test. *n* = 5 mice/group. **b** Hot plate test of *Avil-Cre;Gnaq*^*f/f*^ mice at 48, 50, 53 and 56 °C. Two-way ANOVA followed by Sidak’s multiple comparisons test analysis. *n* = 5 mice/group. **c**, **d** Temperature preference test of *Avil-Cre;Gnaq*^*f/f*^mice at 35 °C/50 °C (**c**) and 35 °C/53 °C (**d**), the time mice spent in the testing area within 5 min was counted. Welch’s *t*-test. *n* = 8 mice/group. **e** Hargreaves test with 35% irradiation intensity of mice received intraplantar injection of PLC antagonist U73122. Unpaired *t*-test. *n* = 8 mice/group. **f** Hargreaves test with 35% irradiation intensity of mice received intraplantar injection of IP3R inhibitor 2-APB. Unpaired *t*-test. *n* = 8 mice/group. **g**–**j** Behavioral tests of mice overexpressing amino acids 780–810 in DRG neurons 8 weeks after i.p. injection of AAV2/9-*Ctrl* or AAV2/9-*aa*^*780*–*810*^ at P0. *n* = 8 mice/group. **g** Hargreaves test with 35% irradiation intensity. Unpaired *t*-test. **h** Hot plate test at 48, 50, 53 and 56 °C. Two-way ANOVA followed by Sidak’s multiple comparisons test analysis. Temperature preference test at 35 °C/50 °C (**i**) and 35 °C/53 °C (**j**), the time for mice spent in the testing zone within 5 min was counted. Unpaired *t*-test. All data are expressed as mean ± s.e.m. *p < 0.05, **p < 0.01, ***p < 0.001, and ****p < 0.0001.

Then, we examined the role of Gαq-PLC-IP3R-Ca^2+^ signaling in noxious heat sensation. We generated *Advillin-Cre*;*Gnaq*^*f/f*^ (*Avil-Cre*; *Gnaq*^*f/f*^) mice, which specifically lacked Gαq expression in primary sensory neurons. The *Avil-Cre*;*Gnaq*^*f/f*^ mice exhibited increased latency in Hargreaves tests and hot plate tests, indicating a deficit in their response to noxious heat stimulation (Fig. 6a, b). Additionally, these mice showed decreased avoidance behavior in response to noxious heat stimulation (Fig. 6c, d and Supplementary Fig. S13a–d), highlighting the essential role of Gαq signaling in thermosensation. Interestingly, *Avil-Cre*;*Gnaq*^*f/f*^ mice show impaired cold and evaporative cooling sensation as well, while mechanosensation remains unaffected (Supplementary Fig. S13e–h). Possibly, within the thermal sensation signaling cascade, Gαq may play a pivotal role in perceiving heat, noxious cold, and cool temperatures. TMC6 selectively modulates the perception of noxious heat through the specific regulation of Gαq, while unidentified receptors, via Gαq, selectively mediate the perception of noxious cold and cool temperatures individually.

To further investigate the involvement of Gαq signaling in thermosensation, we performed intraplantar injection of the PLC antagonist U73122 (100 μM, 10 μL). To exclude the side effects of U73122, U73122’s inactive analog U73343 was taken as a negative control. This resulted in a significant increase in the latency of the Hargreaves test, from 7.825 ± 0.6920 s to 12.28 ± 1.326 s, when exposed to 35% radiant intensity using the Hargreaves apparatus (Fig. 6e). Consistently, intraplantar injection of the IP3R inhibitor 2-APB (200 μM, 10 μL) also led to increased latency in the Hargreaves test (Fig. 6f). Additionally, intraplantar injection of IP3R’s more specific inhibitor Xestospongin C (100 μM, 10 μL) can significantly increase the mice’s latency in Hargreaves test (Supplementary Fig. 14a), supporting the involvement of the Gαq-PLC-IP3R signaling pathway in noxious heat sensation.

To investigate whether TMC6 was involved in Gαq-PLC-IP3R signaling-mediated noxious heat sensation, we specifically disrupted the interaction between TMC6 and Gαq by delivering the polypeptide aa^780-810^ into DRG neurons via intraperitoneal injection of AAV2/9-*aa*^*780*–*810*^ into P0 mice. Mice overexpressing the polypeptide amino acids 780–810 in DRG neurons exhibited significantly increased latencies in the Hargraves test and hot plate tests (Fig. 6g, h). In the temperature preference tests, the polypeptide overexpressing mice displayed significantly reduced avoidance of the testing temperatures of 50 °C and 53 °C (Fig. 6i, j and Supplementary Fig. S14b–e). Notably, the baseline measurements of mechanical pain, evaporative cooling, cold pain, and touch sensitivity remained unaffected (Supplementary Fig. S14f–i), consistent with the phenotype observed in *Tmc6*^−/−^ mice and *Avil-Cre*;*Tmc6*^*f/f*^ mice. Collectively, these findings indicate that the interaction between TMC6 and Gαq is crucial for TMC6-mediated noxious heat sensation.

However, it is important to note that in addition to the defects observed in thermosensation in *Tmc6*^−/−^ mice and when blocking the interaction between TMC6 and Gαq, selective ablation of Gαq in primary sensory neurons resulted in defects in the sensation of cold, cool, and mechanical pain (Supplementary Fig. S13e–h), indicating that the canonical Gαq-PLC-IP3R signaling pathway is also involved in the sensation of cool, cold, and mechanical pain, although the specific GPCRs responsible for these sensations still need to be identified. However, TMC6 confines the Gαq-PLC-IP3R-Ca^2+^ signaling pathway specifically regulates noxious heat sensation (Supplementary Fig. 15).

## Discussion

Thermosensation is a crucial ability shared by all organisms, enabling them to detect and react to variations in environmental temperature for self-protection and benefit. Mammals, including higher primates, have developed specialized structures known as thermoreceptors, which are present in both peripheral and internal organs. These thermoreceptors primarily consist of free nerve endings generated by unmyelinated C-fibers and thin myelinated Aδ-fibers located in the skin or internal organs. Despite numerous studies aimed at unraveling the molecular mechanisms underlying thermosensation, ongoing debates persist regarding the molecular identities and physiological properties of the thermosensors expressed on thermoreceptors. In our recent study, we have identified a novel thermosensor called TMC6, which operates as a Gαq-coupled GPCR-like receptor responsible for mediating thermosensation.

Thermoreceptors are a specific subset of small-sized DRG neurons, characterized by the presence of specific markers such as CGRP or IB4^8^. Interestingly, TMC6 was found to be expressed in DRG neurons without displaying any specific neuronal subtype specificity. This raises the question of how the broadly expressed TMC6 achieves specificity in regulating thermosensation. To address this question, we conducted ISH experiments, which revealed that the downstream signaling component of TMC6, namely Gαq, was selectively present in small-sized DRG neurons. This finding suggests that the restricted expression of Gαq confers specificity to TMC6 in mediating thermosensation. Based on this, we selected small-diameter DRG neurons measuring 10–20 μm for calcium imaging and electrophysiological recordings. Furthermore, when we knocked out TMC6 or blocked the interaction between TMC6 and Gαq using a polypeptide, we observed a specific impact on the perception of noxious heat sensation, while mechanical, cold, and cool sensations remained unaffected. Conversely, when Gαq was ablated or the PLC or inositol trisphosphate receptor (IP3R) pathways were blocked, not only the heat response but also the cool, cold, and mechanical sensations were diminished. These findings indicate that the TMC6-Gαq-PLC-IP3R-Ca^2+^ pathway is responsible for the specificity in mediating thermosensation. Moreover, these findings suggest the existence of other Gαq-coupled GPCRs that specifically respond to mechanical, cool, and cold sensations, and identifying these receptors becomes a critical avenue for future research.

The firing of APs in DRG neurons in response to heat is a critical event in triggering the perception of noxious heat. Prior to AP generation, neurons undergo depolarization, accompanied by Ca^2+^ influx either from the extracellular space or through release from internal stores^9^. The deletion of TMC6 in DRG neurons results in a reduction in both the responsive ratio of DRG neurons and the amplitude of APs elicited by noxious heat, underscoring the essential role of TMC6 in the membrane excitability of DRG neurons. One potential discordance with the DRG neuron excitability is that ΔR/R0 showed no significant difference between WT and *Tmc6*^−/−^ DRG neurons in the presence of extracellular Ca^2+^. An explanation for the lack of a significant reduction in the ΔR/R0 of total recorded *Tmc6*^−/−^ DRG neurons (Fig. 2a–c) could be the simultaneous activation of other noxious sensors (e.g., TRPV1, TRPM3, TRPA1, ANO1, STIM1-ORAI1, and K2P channels) during noxious heat stimulation^8^. Furthermore, the depolarization-induced activation of voltage-gated Ca^2+^ channels may result in a substantial influx of Ca^2+^, diluting the signal originating from TMC6-mediated Ca^2+^ release from the ER. However, when noxious heat-induced Ca^2+^ influx was completely blocked in the absence of extracellular Ca^2+^, the decrease in Ca^2+^ efflux from the ER was unmasked, revealing a discernible reduction in ΔR/R0 in *Tmc6*^−/−^ DRG neurons.

The temperature eliciting TMC6-dependent internal Ca^2+^ release is about 42 °C in HEK293T cells overexpressing TMC6 (Fig. 4e), while it’s about 45 °C in the primarily cultured DRG neuron (Figs. 2a and 3). Multiple factors may result in such discrepancy. Firstly, post-translational modifications, such as phosphorylation, glycosylation, and other post-translational modifications of TMC6 can influence its function, including its activation threshold. Secondly, various modulatory substances, such as endogenous modulators, may either facilitate or inhibit the activation of TMC6, depending on their specific interactions with TMC6 or its downstream components. Thirdly, the different expression levels or the amount of TMC6 localized on cell membrane may also lead to the different activation thresholds of TMC6-overexpressing HEK293T cells and DRG neurons.

One limitation of this study is the current lack of understanding of how TMC6-mediated Ca^2+^ release from the ER regulates noxious heat-elicited APs. We used to explore the potential downstream effectors of TMC6-mediated ER calcium release, the results showed that TRPA1, TRPV1, TRPM3, and total sodium current were not affected by the ablation of TMC6 (Supplementary Fig. 7b–l). However, multiple mechanisms for TMC6-Gαq-PLC-IP3R-Ca^2+^ signaling-mediated DRG neuron firing can still be predicted from studies on signaling from endogenous Gq-coupled GPCRs. The activation of Gαq-PLC can reduce the M-current^39^, which is a PIP2-sensitive K^+^ conductance mediated by Kcnq channels^40^. The activated PLC converts PIP2 to IP3, and reduced PIP2 decreases M-current in Kcnq-expressing cells, consequently increasing neuronal excitability^41^. A paper with similar conclusions came out while this manuscript was in revision^42^. Previous studies also showed that the release of Ca^2+^ from ER can activate the Na^+^/Ca^2+^ exchanger (NCX)^43^, which is electrogenic and can lead to a depolarizing inward current when internal Ca^2+^ is elevated, complementing depolarization from M-current suppression^41,44,45^. We checked the expression of KCNQs and NCXs in DRG neurons in the single-cell RNA sequencing database. Interestingly, KCNQ2, KCNQ5, KCNQ1OT1, NCX1 (Gene: *Slc8a1*), and NCX3 (Gene: *Slc8a3*) show high expression in DRG neurons (https://linnarssonlab.org/drg/), which are potential downstream effectors of TMC6-Gαq-PLC-IP3R-Ca^2+^ signaling. We are actively investigating the molecular mechanisms and the downstream effectors of TMC6-Gαq-PLC-IP3R-Ca^2+^ signaling.

To summarize, our study offers valuable insights into the molecular properties of thermosensors and the intricate mechanisms underlying thermosensation. The discovery of TMC6 as a Gαq-coupled GPCR-like receptor that specifically responds to noxious heat stimulation expands our understanding of the diversity of thermosensor molecules. Additionally, these findings highlight the intricate nature of thermosensation, where multiple signaling pathways and receptors likely work in tandem to enable organisms to detect and respond to temperature changes in their environment. Further research in this field will undoubtedly illuminate the fascinating intricacies of thermosensation and its significance across various organisms.

## Materials and methods

### Animals

*Tmc6*^−/−^ mice and *Tmc6*^*f/f*^ mice were generated with CRISPR/Cas9 system by Cyagen Biosciences Inc. *Gnaq*^*f/f*^ mice were ordered from GemPharmatech Co. Ltd. *Advillin-cre* mice and *Trpv1*^−/−^ mice were provided by Dr. Zhenzhong Xu’s lab at Zhejiang University. C57BL/6 WT mice were purchased from Shanghai Lingchang Biological Technology Company. Mice were group-housed and bred in Fudan University animal facilities with 12-h/12-h light/dark cycle at 22 ± 1 °C and free access to food and water. All the animal procedures were approved by the Animal Care and Use Committee of the Institutes of Brain Science of Fudan University and were conducted by the National Institutes of Health Guide for the Care and Use of Laboratory.

### Intraplantar injection

In order to assess the effects of peripheral administration of the inhibitors, U73343 (MCE, Cat# HY-108630), U73122 (Selleck, Cat# S8011), 2-APB(Tocris Bioscience, Cat# 1224), and Xestospongin C(MCE, Cat# HY-103312) were injected into the plantar surface of a hind paw of 8-week old mice with a 10 μL volume.

### Intraperitoneal injection of AAV virus in P0 mice

The AAV2/9-*aa*^*780–810*^ (Shanghai Taitool Bioscience Co. Ltd., 5 × 10^12^ v.g./mL, 10 μL) or AAV2/9-*Cre* (Shanghai Jikai gene, 5 × 10^12^ v.g./mL, 10 μL) was intraperitoneally injected in P0 mice. Firstly, the urine was drained from the bladder by gentle pressure on the abdomen of the neonatal mice, then the virus was injected intraperitoneally using a microliter syringe (Shanghai Gaoge). After the injection was completed, the injection site was pressed for 2 min to prevent the virus from leaking out. This method of injection led to the highly selective infection of AAV2/9 in ganglionic neurons, but few in skin cells, spinal neurons, or cerebral neurons, which allowed us to specifically overexpress amino acids 780–810 or Cre enzyme in DRG neurons.

### fMRI

Ten WT mice and ten *Tmc6*^−/−^ mice were included in the fMRI study. Mice were initially induced to anesthesia by 4% isoflurane and a bolus injection of 0.016 mg/kg dexmedetomidine intraperitoneally. Animals were subsequently transferred to the MRI bed with heating systems and fixed by bite and ear bars. Isoflurane level was then gradually reduced and maintained at 0.6% with the continuous subcutaneous infusion of dexmedetomidine at a rate of 0.096 mg/kg/h. During the entire experiment, animals were maintained at a light and stable level of anesthesia and the rectal temperature was monitored and kept at 37.5 ± 0.5 °C (SA Instruments). Detailed procedures were similar with our previous fMRI reports^46,47^. A contact heat evoked potentials thermode (Medoc, Israel) was attached to the left hind paw to deliver the noxious heat stimuli. Two trials were performed on each mouse, and each trial consisted of seven heat blocks (48 °C, 20 s) with 140 s interstimulus interval (35 °C). In total, 550 images were collected for each trial.

MRI experiments were performed on a 9.4 T animal scanner with a 30 cm diameter bore (uMR 9.4 T, United Imaging Life Science Instrument, China). An 86-mm diameter transmit volume coil and a custom single-loop surface coil were used. The fMRI images were acquired using a gradient-echo echo-planar imaging (GE-EPI) sequence with the following parameters: repetition time (TR) = 2000 ms; echo time (TE) = 16 ms; flip angle (FA) = 60°–70°; FOV = 19 × 15 mm^2^; matrix size = 96 × 76; slice number = 28; slice thickness = 0.35 mm; 550 repetitions. The fast spin echo (FSE) sequence was used to obtain the anatomical T2-weighted images with the following parameters: TR = 3000 ms; TE = 38.64 ms; echo train length (ETL) = 11; matrix size = 256 × 202; 4 averages; the geometric parameters (i.e., FOV, slice number and thickness) were the same as the GE-EPI sequence.

All the preprocessing and analysis of fMRI data were conducted on Analysis of Functional NeuroImages (AFNI) (NIH, USA) software and custom scripts in MATLAB (MathWorks, USA). The preprocessing step consisted of slice timing, motion correction, registration to a set of mean anatomical images generated by iterative registering the structure images of all twenty animals, spatial smoothing with a 0.4 mm full width at half maximum (FWHM) Gaussian kernel, and temporal filtering at the frequency of 0.001–0.2 Hz. The hemodynamic response function was used to generate a regressor, which was used for estimating the blood oxygen level-dependent (BOLD) response to stimulation by ordinary least squares. Further, in order to obtain the group activation maps, unpaired Student’s *t*-test was applied to calculate the *t*-value maps of each group. The statistical significance level was set to *p* < 0.05, with multiple comparisons corrected by false discovery rate. The statistical activation maps were superimposed on the mean structural images with a minimum cluster size of 40 voxels. For quantificationally comparing the differences in BOLD response to heat stimulation between the two groups, seven brain regions: ACC, Amyg, CPu, Ins, S1, S2, and thalamus, which respond to thermal stimuli were selected and the corresponding regions of interests (ROIs) were identified according to a mouse atlas (Allen Institute, USA). The signal changes of each ROI were calculated based on the extracted fMRI time courses and the data exceeding the criterion of mean + 3 × SD were excluded. A non-parametric test (Mann–Whitney *U*) was used because the test statistic did not follow a normal distribution.

### Computational simulations

The structural models of TMC6 and TMC6-Gαq complex were generated by AlphaFold2 and AlphaFold multimer, respectively^48^. To embed TMC6 into the membrane model, TMHMM-2.0 and OPM2 were applied to predict the transmembrane regions and their orientation^49^. CHARMM-GUI server was used to integrate the structural and orientation information and build the all-atom model for molecular dynamics simulations on Gromacs 2019.6^48,50^. Briefly, the TMC6–Gαq protein complex was positioned in the bilayers of cholesterol, POPC, and POPE phospholipids, then solvated with TIP3P waters and ions (~392,000 atoms) in a box of 153 × 153 × 177 Å^3^. The Nose-Hoover thermostat (303.15 K or 323.15 K) and Parrinello-Rahman isotropic NPT ensemble were adopted with h-bonds LINCS constraints. After equilibration, three 100-ns trajectories were performed independently. The conformations of TMC6–Gαq were clustered throughout the trajectory and analyzed for the distance between TMC6 and Gαq using Gromacs tools (gmx distance).

### Quantification and statistical analysis

Data normality was initially evaluated via Shapiro–Wilk and Kolmogorov–Smirnov tests, considering data normally distributed at *p* > 0.05. Variance homogeneity was assessed using the *F* test, with *p* > 0.05 indicating homoscedasticity. For normally distributed, related samples with equal variances, paired *t*-tests were conducted. Conversely, independent samples with equal variances were analyzed using unpaired *t*-tests, and those with unequal variances via Welch’s *t*-test. Data subjected to log2 transformation, following non-normal distribution, adhered to these parametric tests. Non-transformable, non-normal data were analyzed using the Mann–Whitney *U*-test. One-way ANOVA was deployed to determine mean differences among three or more distinct groups relative to a single dependent variable, complemented by Dunnett’s multiple comparisons test for assessing differences between each experimental group and a control group. For non-parametric testing of one-factor multiple group comparisons, we use the Kruskal–Wallis test followed by Dunn’s multiple comparisons test analysis. To assess the effects of two independent variables on a dependent variable and their interaction, Two-way ANOVA was applied, followed by Sidak’s multiple comparisons test for precise adjustment in the context of multiple comparisons. Statistical methodologies are detailed in figure legends, with results expressed as mean ± s.e.m. Significance levels are indicated as *p < 0.05, **p < 0.01, ***p < 0.001 and ****p < 0.0001.

### Supplementary information

Supplementary Materials
