# Supplementary material for: TMC6 functions as a GPCR-like receptor to sense noxious heat via Gαq signaling
Source: Cell Discov. 2024 Jun 18;10:66. doi: 10.1038/s41421-024-00678-9 (PMC11183229; doi:10.1038/s41421-024-00678-9)
Supplement: Supplementary file 1 — Supplementary Materials [file 41421_2024_678_MOESM1_ESM.pdf]

Supplementary Figures and Figure legends

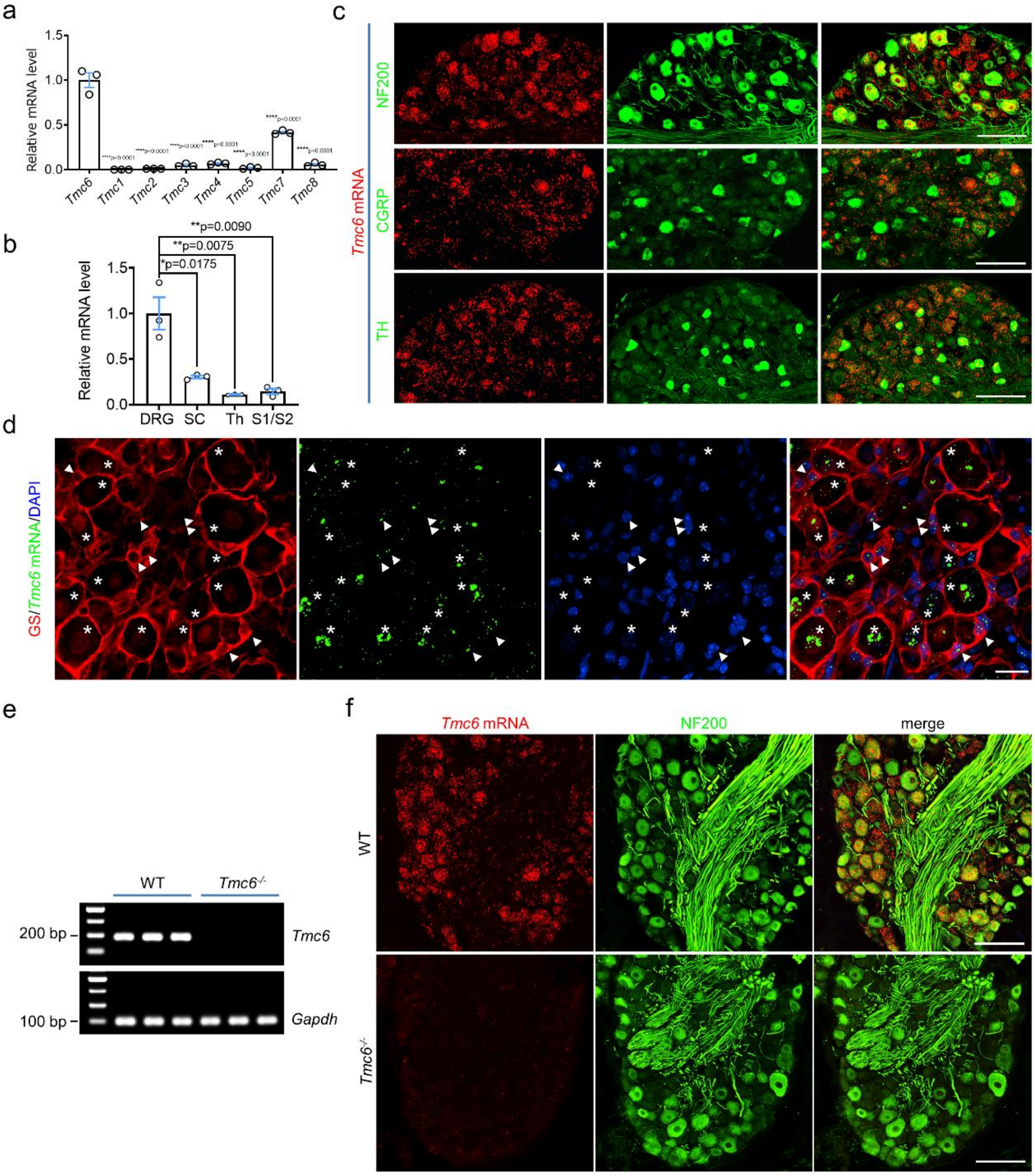

Supplementary Fig. S1 The expression and knockout efficiency of TMC6 in DRG.

a Quantitative real-time PCR (qRT-PCR) was employed to assess the expression of *Tmc1*-*Tmc8* in mouse DRG, One-way ANOVA followed by Dunnett's multiple comparisons test analysis. n = 3.

b qPCR tests the expression of *Tmc6* in DRG, spinal cord (SC), thalamus (Th) and sensory cortex (S1/S2), unpaired t-test. n = 3.

c ISH with probe against mouse *Tmc6* (Red) combined with immunohistochemistry (IHC) with antibody against Neurofilament 200 (NF200), Calcitonin gene-related

peptide (CGRP), or with FITC-conjugated Isolectin B4. Scale bars, 100 μm.

d ISH with probe targeting mouse *Tmc6* (Green) combined with IHC to test the expression of *Tmc6* in satellite glial cell (GS^+, Red， arrowhead) and DRG neuron (star). Scale bar, 20 μm.

e Reverse transcription-PCR (RT-PCR) was used to test the knockout efficiency of *Tmc6* in DRG, with primers spanning 1570 to 1747 nt of full length *Tmc6* mRNA. The band sizes for *Tmc6* and *Gapdh* are 178 bp and 95 bp respectively.

f ISH examined the expression of *Tmc6* in DRG of WT mice and $Tmc6^{-/-}$ mice. Scale bar, 100 μm.

All data are expressed as mean ± s.e.m. $^*p < 0.05$, $^{**}p < 0.01$, and $^{****}p < 0.0001$.

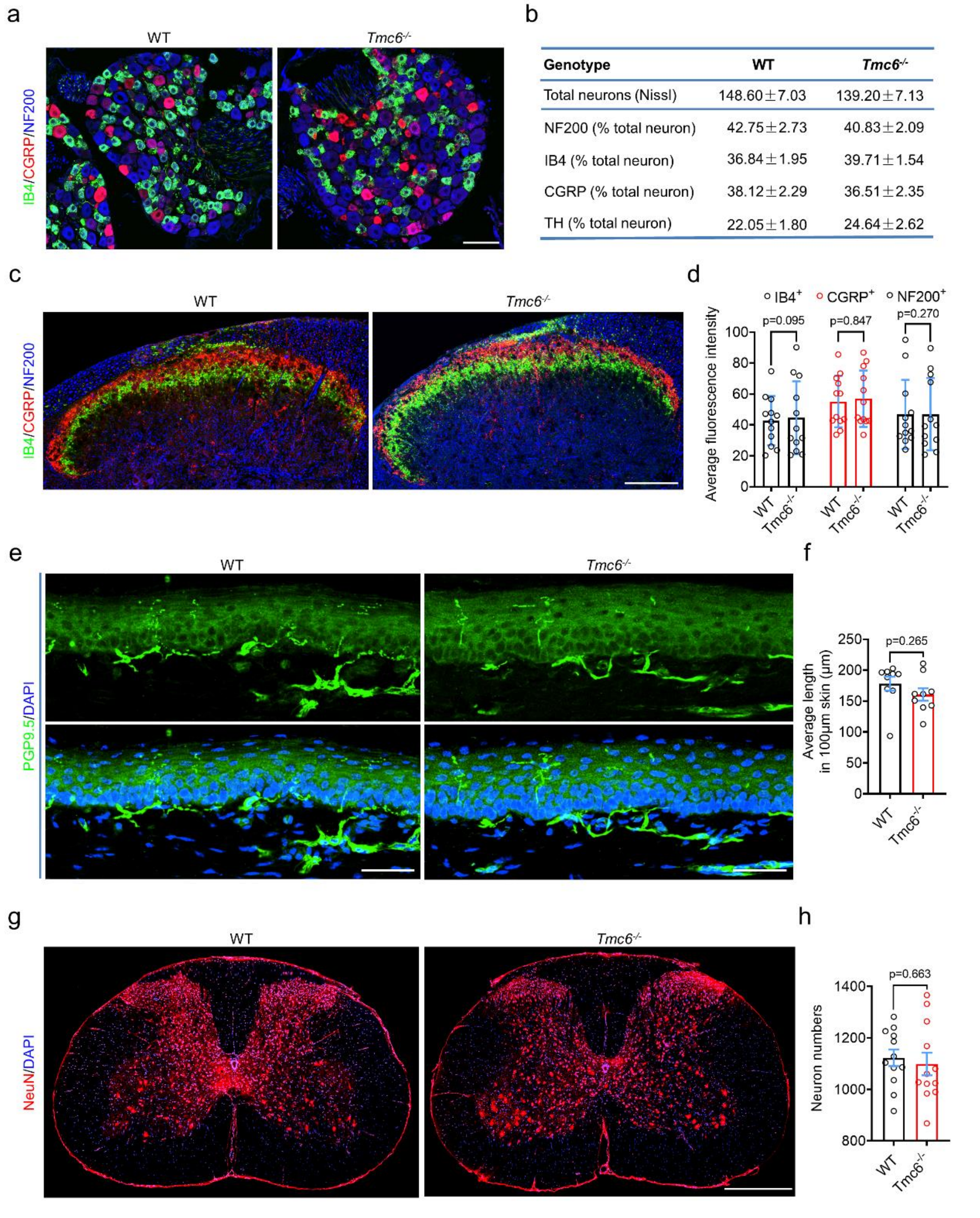

Genotype	WT	*Tmc6*$^{-/-}$
Total neurons (Nissl)	148.60±7.03	139.20±7.13
NF200 (% total neuron)	42.75±2.73	40.83±2.09
IB4 (% total neuron)	36.84±1.95	39.71±1.54
CGRP (% total neuron)	38.12±2.29	36.51±2.35
TH (% total neuron)	22.05±1.80	24.64±2.62

Supplementary Fig. S2 *Tmc6*$^{-/-}$ mice have normal neurodevelopment.

a Triple staining of IB4 (green), CGRP (red), and NF200 (blue) on DRG sections from WT mice or *Tmc6*$^{-/-}$ mice separately. Scale bar, 100 μm.

b Quantification of the percentages of CGRP, IB4, NF200, and TH neurons in DRG of WT and *Tmc6*$^{-/-}$ mice separately. n = 15-16 DRGs from 3 mice/group.

c Immunostaining of IB4, CGRP, and NF200 on spinal cord sections from WT mice or

Tmc6$^{-/-}$ mice. Scale bar, 100 µm.

d Quantification of average fluorescence intensity of IB4, CGRP, NF200 on spinal cord sections from WT and *Tmc6*$^{-/-}$ mice. n = 12 spinal cord sections from 3 mice/group.

e Double-staining with pan neuronal marker PGP9.5 and DAPI in the skin of hind paw of WT mice or *Tmc6*$^{-/-}$ mice. Scale bar, 50 µm.

f Quantification of the total length of PGP9.5^{+} nerve fiber per 100 µm on skin sections from WT and *Tmc6*$^{-/-}$ mice. n = 9 skin sections from 3 mice/group.

g Double-staining of NeuN and DAPI on spinal cord sections from WT mice or *Tmc6*$^{-/-}$ mice. Scale bar, 500 µm.

h Quantification of the number of neurons on spinal cord sections from WT and *Tmc6*$^{-/-}$ mice. n = 12 spinal cord sections from 3 mice/group.

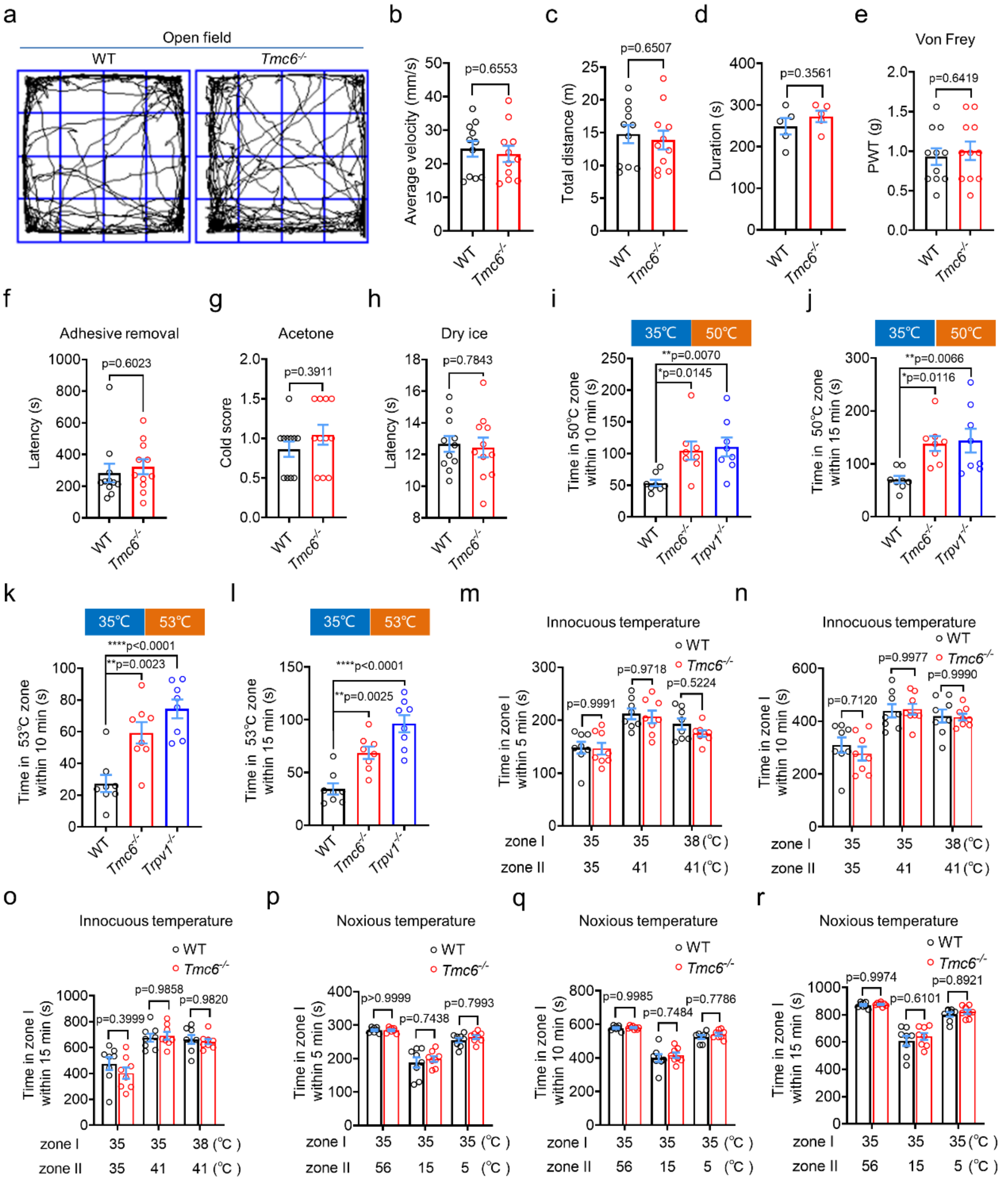

Supplementary Fig. S3 Motor function and somatosensation of *Tmc6*$^{-/-}$ mice.

a-c Open field test of WT and *Tmc6*$^{-/-}$ mice. n = 11 mice/group **a** The representative motion trajectory diagram of WT and *Tmc6*$^{-/-}$ mice in a 40 cm × 40 cm × 40 cm box. **b** Statistics of average velocity of WT mice and *Tmc6*$^{-/-}$ mice. **c** Statistics of total distance for WT mice and *Tmc6*$^{-/-}$ mice moved. Unpaired t-test.

d Statistics of rotarod test of WT mice and *Tmc6*$^{-/-}$ mice. Unpaired t-test. n = 5 mice/group.

e-h Behavioral tests of WT mice and *Tmc6*$^{-/-}$ mice. n = 11 mice/group. **e** Von Frey test for baseline of mechanical pain. Unpaired t-test. **f** Adhesive removal test for touch sensitivity. Unpaired t-test. **g** Acetone test for evaporative cooling sensitivity. Mann-Whitney U test. **h** Dry ice test for noxious cold sensitivity. Unpaired t-test.

i, **j** Temperature preference test of WT, *Tmc6*$^{-/-}$, *Trpv1*$^{-/-}$ mice, reference temperature was set at 35°C, and testing temperature was set at 50°C, the time mice spent in testing area within 10 min (**i**) and 15 min (**j**) were counted. One-way ANOVA followed by Dunnett's multiple comparisons. n = 8 mice/group.

k, **l** Temperature preference test of WT, *Tmc6*$^{-/-}$, *Trpv1*$^{-/-}$ mice, reference temperature was set at 35°C, and testing temperature was set at 53°C, the time mice spent in testing area within 10 min (**k**) and 15 min (**l**) were counted. One-way ANOVA followed by Dunnett's multiple comparisons. n = 8 mice/group.

m-o Temperature preference test of WT mice and *Tmc6*$^{-/-}$ mice under innocuous temperatures, including 35°C-35°C, 35°C-41°C, 38°C-41°C separately. The time mice spent in zone I within 5 min (**m**), 10 min (**n**) and 15 min (**o**) were shown. Two-way ANOVA followed by Sidak's multiple comparisons. n = 8 mice/group.

p-r Temperature preference test of WT mice and *Tmc6*$^{-/-}$ mice under noxious temperatures, including 35°C-56°C, 35°C-15°C, 35°C-5°C separately. The time mice spent in zone I within 5 min (**p**), 10 min (**q**) and 15 min (**r**) were shown. Two-way ANOVA followed by Sidak's multiple comparisons. n = 8 mice/group.

All data are expressed as mean ± s.e.m. *$p < 0.05$, **$p < 0.01$, and ****$p < 0.0001$.

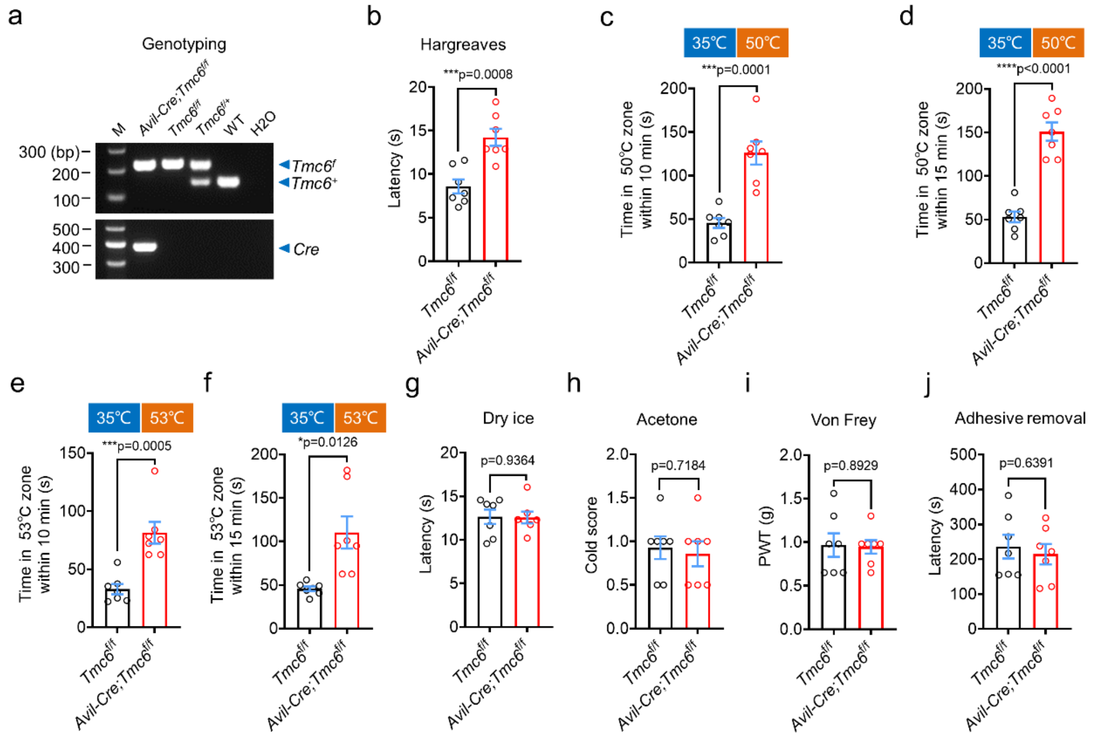

Supplementary Fig. S4 Genotyping and behavioral tests of *Avil-Cre;Tmc6*$^{f/f}$ mice.

a Genotyping of *Avil-Cre;Tmc6*$^{f/f}$ mice. Homozygotes: single band at 210 bp; Heterozygotes: two bands at 210 bp and 136 bp separately; WT allele: one band at 136 bp; *Avil-Cre* allele: one band at approximately 400 bp.

b Hargreaves test with 35% irradiation intensity of *Avil-Cre;Tmc6*$^{f/f}$ mice. Unpaired t-test. n = 7 mice/group.

c, **d** Temperature preference test of *Avil-Cre;Tmc6*$^{f/f}$ mice, reference temperature was set at 35℃, and testing temperature was set at 50℃, the time for mice spent in testing area within 10 min (**c**) and 15min (**d**) were shown. Unpaired t-test. n =7 mice/group.

e, **f** Temperature preference test of *Avil-Cre;Tmc6*$^{f/f}$ mice, reference temperature was set at 35℃, and testing temperature was set at 53℃, the time mice spent in testing area within 10 min (**e**) and 15min (**f**) were shown. (**e**) Unpaired t-test. n = 7 mice/group. (**f**) Welch's t-test. n = 7 mice/group.

g Dry ice test of *Avil-Cre;Tmc6*$^{f/f}$ mice for noxious cold sensitivity. Unpaired t-test. n = 7 mice/group.

h Acetone test of *Avil-Cre;Tmc6*$^{f/f}$ mice for evaporative cooling sensitivity. Unpaired t-

test. n = 7 mice/group.

i Von Frey test of *Avil-Cre;Tmc6*$^{f/f}$ mice for baseline of mechanical pain. Unpaired t-test. n = 7 mice/group.

j Adhesive removal test of *Avil-Cre;Tmc6*$^{f/f}$ mice for touch sensitivity. Unpaired t-test. n = 7 mice/group.

All data are expressed as mean ± s.e.m. *$p < 0.05$, ***$p < 0.001$ and ****$p < 0.0001$.

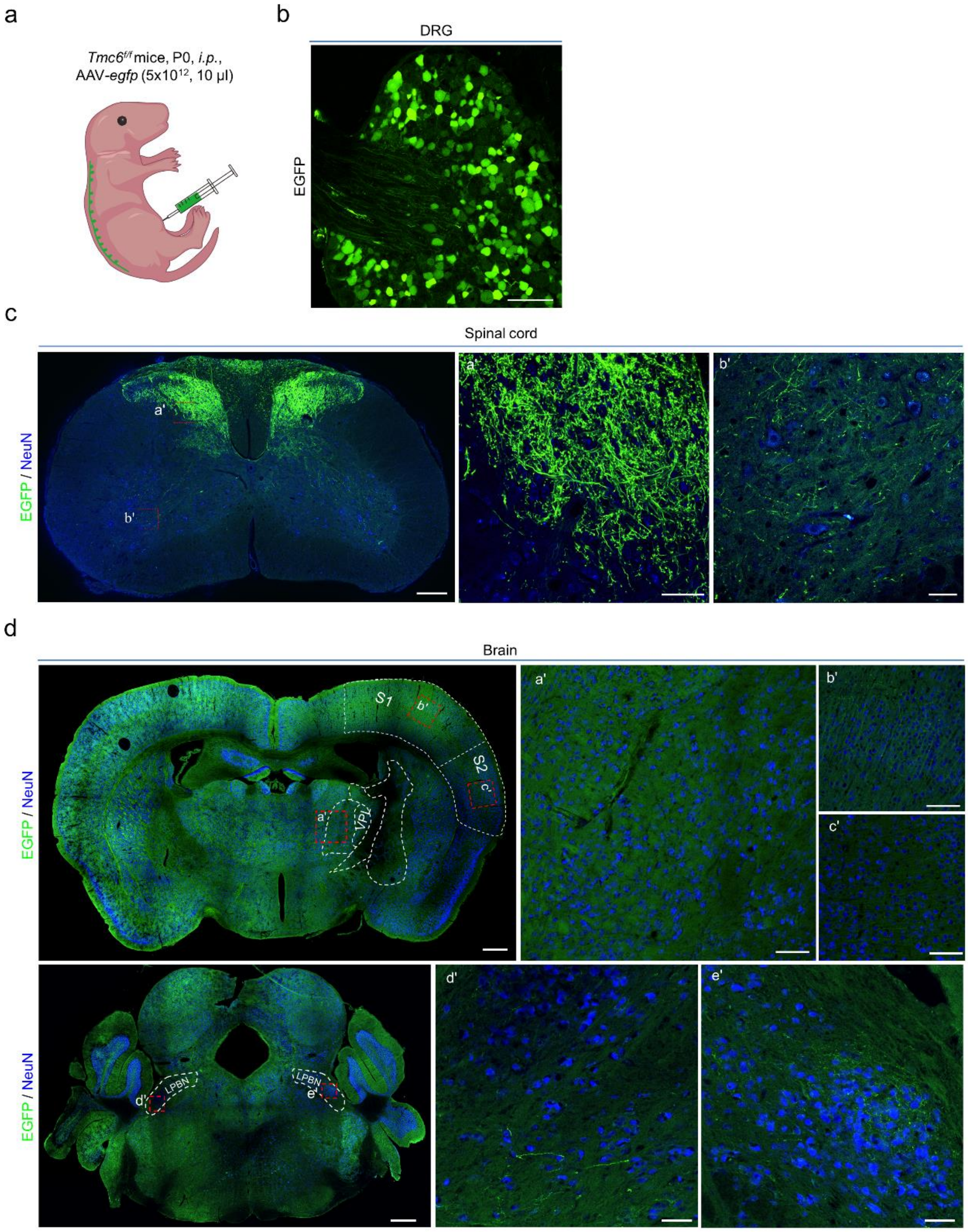

Supplementary Fig. S5 Intraperitoneal injection (i.p.) of AAV in P0 mice selectively infects DRG neurons.

a Schematic diagram illustrating intraperitoneal injection of AAV-*Cre-egfp* in *Tmc6*$^{f/f}$ mice at P0.

b Representative image showing infection of AAV-*Cre-egfp* in DRG. Scale bar, 100 µm.

c Immunostaining with pan-neuronal marker NeuN on spinal cord sections from *Tmc6*$^{f/f}$ mice i.p. injected with AAV-*Cre-egfp* at P0. Scale bar in the left panel is 200 µm, in middle panel and right panel are 50 µm.

d Immunostaining with pan-neuronal marker NeuN on brain sections from *Tmc6*$^{f/f}$ mice i.p. injected with AAV-*Cre-egfp* at P0. Scale bars in left panels are 500 µm, in **a'-c'** are 100 µm, and in **d'-e'** are 50 µm.

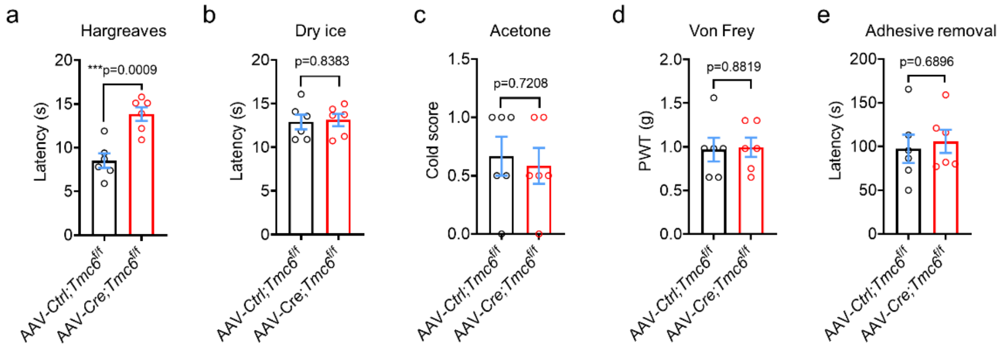

Supplementary Fig. S6 Behavioral test of AAV-*Cre;Tmc6*$^{f/f}$ mice.

a Hargreaves test with 35% irradiation intensity of AAV-*Cre;Tmc6*$^{f/f}$ mice. Unpaired t-test. n = 6 mice/group.

b Dry ice test of AAV-*Cre;Tmc6*$^{f/f}$ mice. Unpaired t-test. n = 6 mice/group.

c Acetone test of AAV-*Cre;Tmc6*$^{f/f}$ mice. Unpaired t-test. n = 6 mice/group.

d Von Frey test of AAV-*Cre;Tmc6*$^{f/f}$ mice. Unpaired t-test. n = 6 mice/group.

e Adhesive removal test of AAV-*Cre;Tmc6*$^{f/f}$ mice. Unpaired t-test. n = 6 mice/group. All data are expressed as mean ± s.e.m. ***$p < 0.001$.

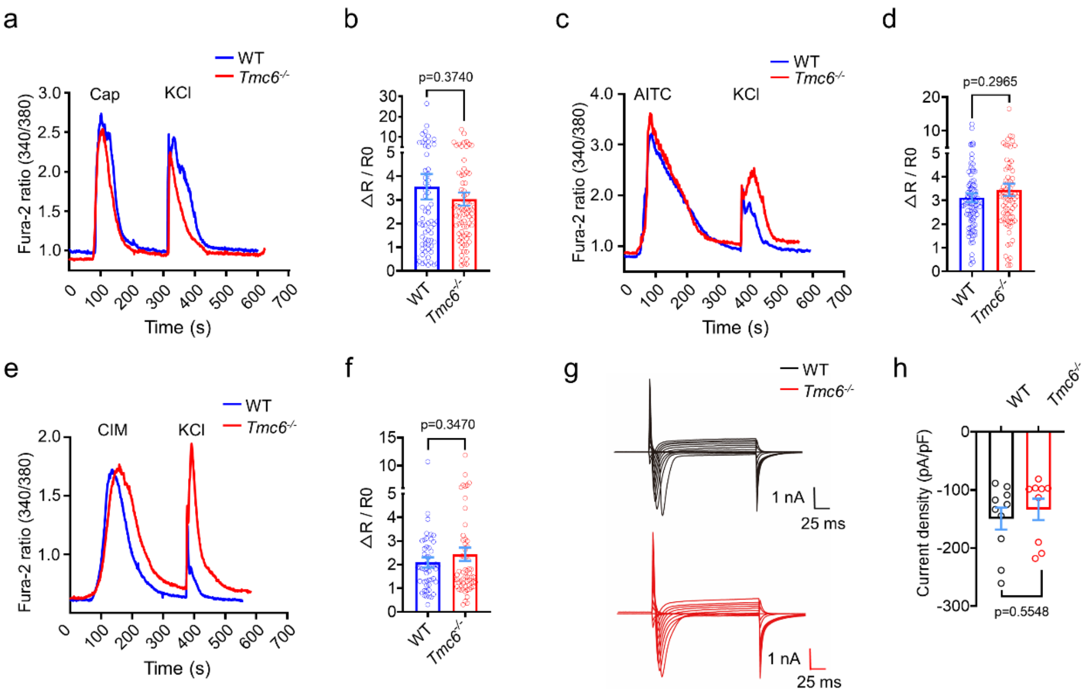

Supplementary Fig. S7 TMC6 affects neither TRP channels nor whole cell sodium current.

a, **b** Fura-2 ratiometric calcium imaging of cultured WT and *Tmc6*$^{-/-}$ DRG neurons in response to 1 µM capsaicin. **a** Representative traces of capsaicin-induced ratio changes in cultured DRG neurons from WT mice and *Tmc6*$^{-/-}$ mice. **b** Statistics of capsaicin - induced ΔR/R0, showing as bar graphs combined with scatter dot plots. Unpaired t-test. n = 65-74 neurons/group, from 5 DRG neuron preparations.

c, **d** Fura-2 ratiometric calcium imaging of cultured WT and *Tmc6*$^{-/-}$ DRG neurons in response to 200 µM Allyl isothiocyanate (AITC). **c** Representative traces of AITC-induced ratio changes in cultured DRG neurons from WT mice and *Tmc6*$^{-/-}$ mice. **d** Statistics of AITC-induced ΔR/R0, showing as bar graphs combined with scatter dot plots. Unpaired t-test, n = 74-86 neurons/group, from 5 DRG neuron preparations.

e, **f** Fura-2 ratiometric calcium imaging of cultured WT and *Tmc6*$^{-/-}$ DRG neurons in response to 1 µM CIM0216. **e** Representative traces of CIM0216-induced Fura2 ratio changes in cultured DRG neurons from WT mice and *Tmc6*$^{-/-}$ mice. **f** Statistics of CIM0216-induced ΔR/R0, showing as bar graphs combined with scatter dot plots.

Unpaired t-test. n = 51-61 neurons/group, from 2 DRG neuron preparations.

g, **h** The sodium current of cultured WT and *Tmc6*$^{-/-}$ DRG neurons with holding potential ranging from -80 mV to +60 mV. **g** Representative traces of total sodium currents were recorded in DRG neurons from WT (black lines) mice and *Tmc6*$^{-/-}$ (red lines) mice individually. **h** Quantitative analysis of sodium currents. Unpaired t-test. n = 9-10 neurons/group.

All data are expressed as mean ± s.e.m.

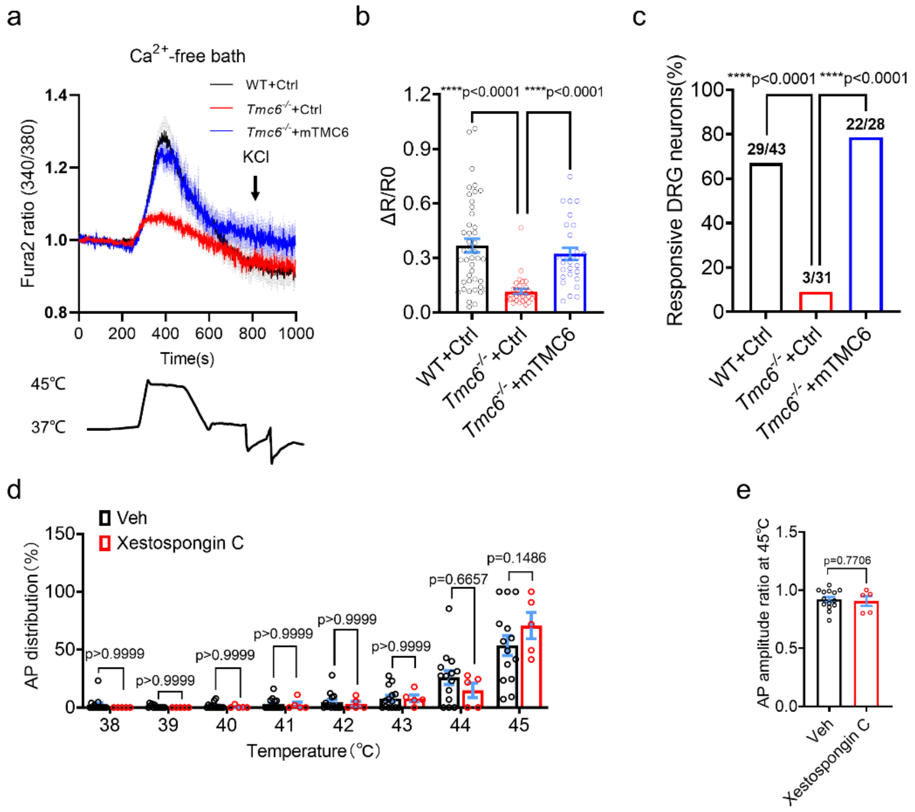

Supplementary Fig. S8 TMC6-mediated internal Ca^{2+} release essentially regulates noxious heat-elicited action potential discharge.

a-c Fura-2 ratiometric calcium imaging of cultured *Tmc6*$^{-/-}$ DRG neurons electroporated with mTMC6 plasmid. **a** Average traces of normalized Fura-2 ratios in WT+Ctrl, *Tmc6*$^{-/-}$+Ctrl, and *Tmc6*$^{-/-}$+mTMC6 DRG neurons. **b** Statistics of heat-induced ΔR/R0. Kruskal-Wallis test followed by Dunn's multiple comparisons test analysis. **c** The responsive percentage of DRG neurons to heating in Ca^{2+}-free bath. $\chi 2$ test. n = 28-43 neurons, from 4 DRG neuron preparations.

d The distribution of heat-induced APs at each degree in Veh group and Xestospongin C-treated group (5 μM), n = 5-14 neurons/group.

e Normalized amplitude of heat-induced APs at 45℃, n=5-14 neurons/group.
All data are expressed as mean ± s.e.m. ****p < 0.0001.

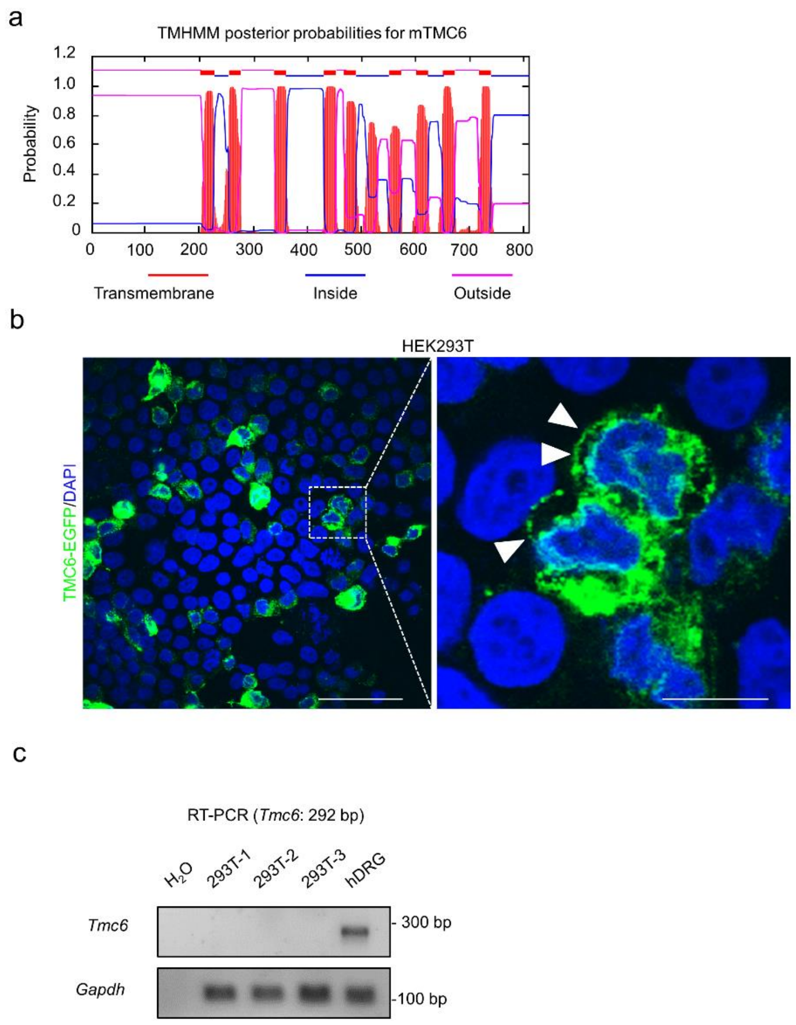

Supplementary Fig. S9 TMC6 localizes on the cell membrane.

a Prediction of mTMC6 transmembrane regions. The ten red bars corresponded to ten transmembrane helixes (the amino acid numbers were indicated at X axis and the probability of each residue belonging to either transmembrane, cytosolic/inside, or outside region was indicated at Y axis).

b HEK293T cells overexpressing *pegfp*-N3-2HA-m*Tmc6*, EGFP (green), DAPI (blue). Scale bars, 50 μm and 10 μm respectively.

c RT-PCR tested the endogenous expression of TMC6 in HEK293T cells, with H_2O as the negative control and human DRG as the positive control. *Tmc6* allele: one band at 292 bp, *Gapdh* allele: one band at 115 bp.

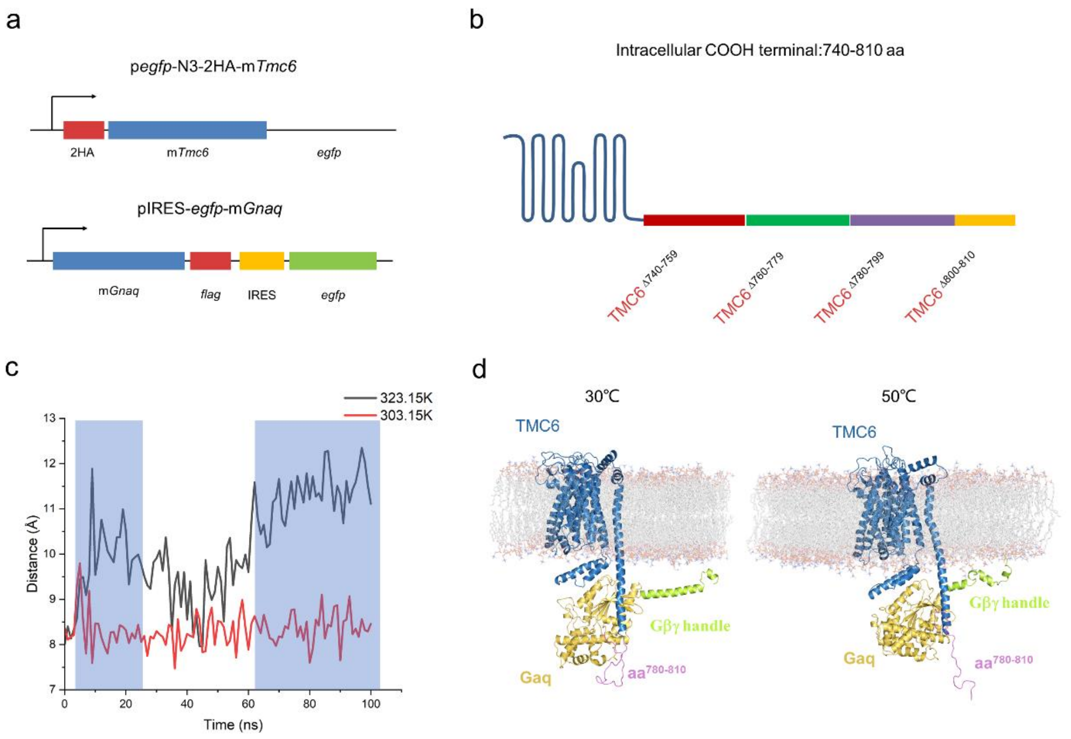

Supplementary Fig. S10 The dynamic interaction between TMC6 and Gαq.

a Schematic diagram of p*egfp*-N3-2HA-m*Tmc6* (upper panel) and pIRES-*egfp*-m*Gnaq* (lower panel).

b Schematic diagram of TMC6 truncations.

c Average distance between TMC6 and Gαq throughout the 100 ns trajectory at 323.15K (50 ℃, black) and 303.15K (30 ℃, red) indicating higher possibility for Gαq to dissociate (blue shaded region).

d Simulated conformations of the TMC6-Gαq complex at different temperatures (Left: 30˚C Right: 50˚C). The COOH-terminal of TMC6 (Ser^{780} to P^{810}, purple color) flipped away from Gαq (yellow color) at elevated temperature and the Gβγ handle (green color) of Gαq featured deformation with respect to the overall Gαq fold.

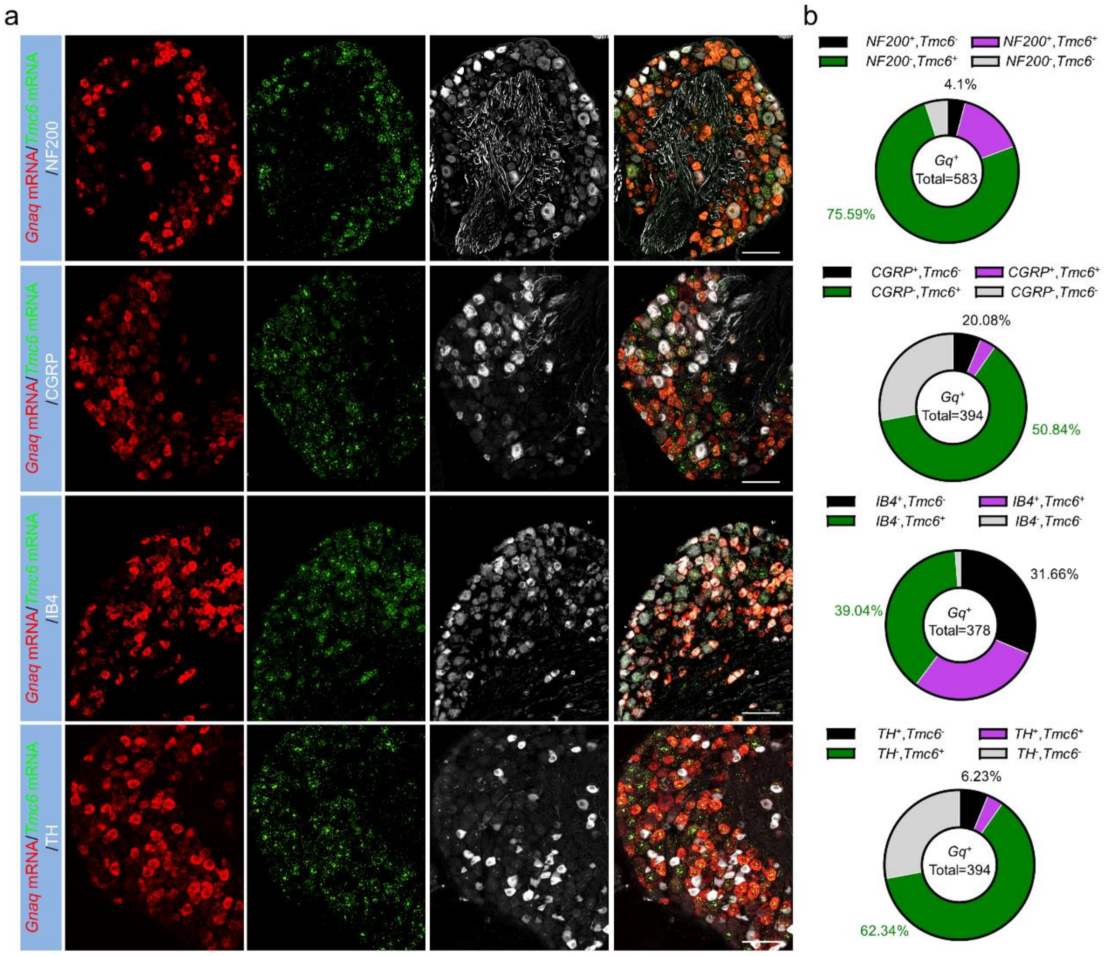

Supplementary Fig. S11 Cell-type profiling of *Gnaq* expression in mouse DRG.

a In situ hybridization (ISH) using probes targeting mouse *Gnaq* and *Tmc6*, combined with immunostaining for markers of distinct DRG neuron subtypes, including medium- or large-sized neurons (NF200), non-peptidergic neurons (IB4), peptidergic neurons (CGRP), and C-fiber low-threshold mechanoreceptors (TH). Scale bar, 100 μm.

b Quantitative analysis of the colocalization of *Gnaq*, *Tmc6*, and specific markers: NF200 (583 *Gnaq*$^+$ neurons from 3 male mice), CGRP (394 *Gnaq*$^+$ neurons from 3 mice), IB4 (378 *Gnaq*$^+$ neurons from 3 male mice), and TH (394 *Gnaq*$^+$ neurons from 3 male mice).

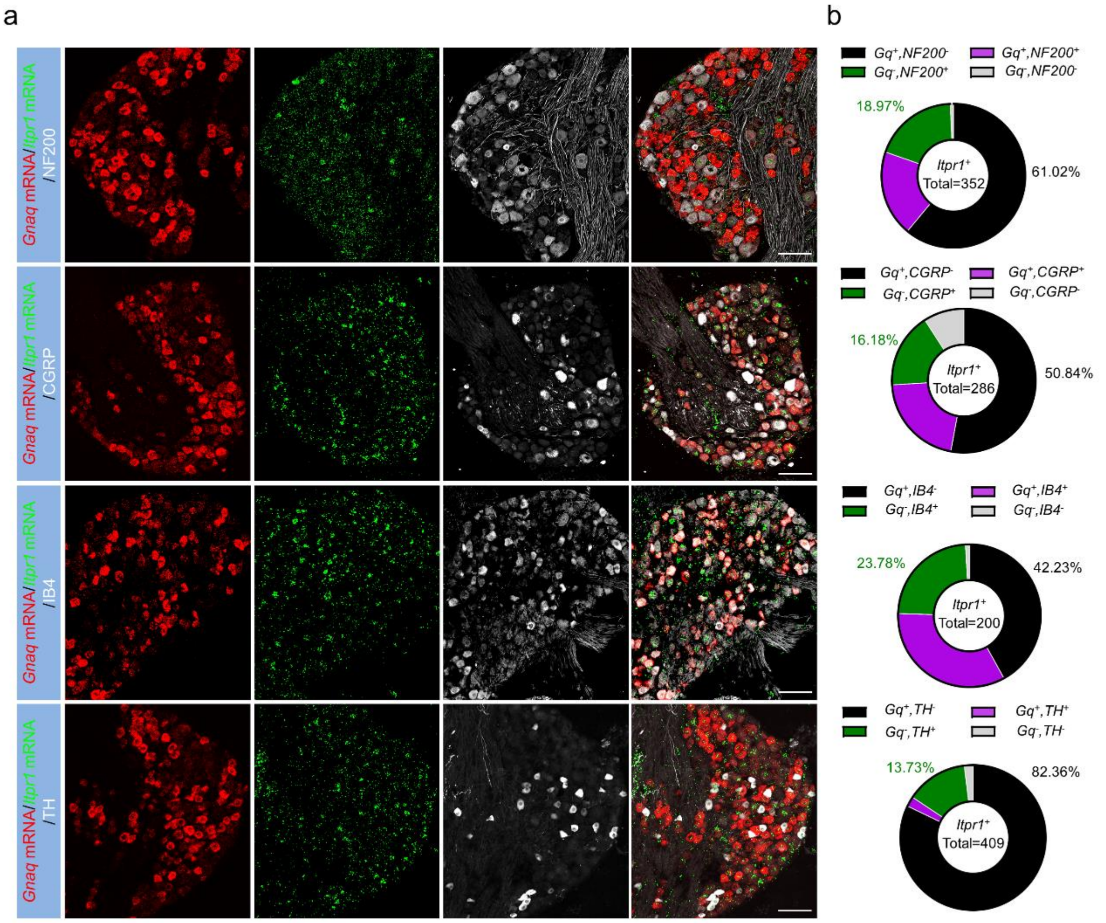

Supplementary Fig. S12 Cell-type profiling of *Ip3r1* in DRG.

a In situ hybridization (ISH) using probes targeting mouse *Gnaq* and mouse *Ip3r1* combined with immunostaining for specific markers of distinct DRG neuron subtypes, including large-sized neurons (NF200), non-peptidergic neurons (IB4), peptidergic neurons (CGRP), and C-fiber low-threshold mechanoreceptors (TH). Scale bar, 100 µm.

b Quantitative analysis of the colocalization of *Gnaq*, *Ip3r1*, and specific markers: NF200 (352 *Ip3r1*$^+$ neurons from 3 male mice), CGRP (286 *Ip3r1*$^+$ neurons from 3 mice), IB4 (200 *Ip3r1*$^+$ neurons from 3 male mice), and TH (409 *Ip3r1*$^+$ neurons from 3 mice).

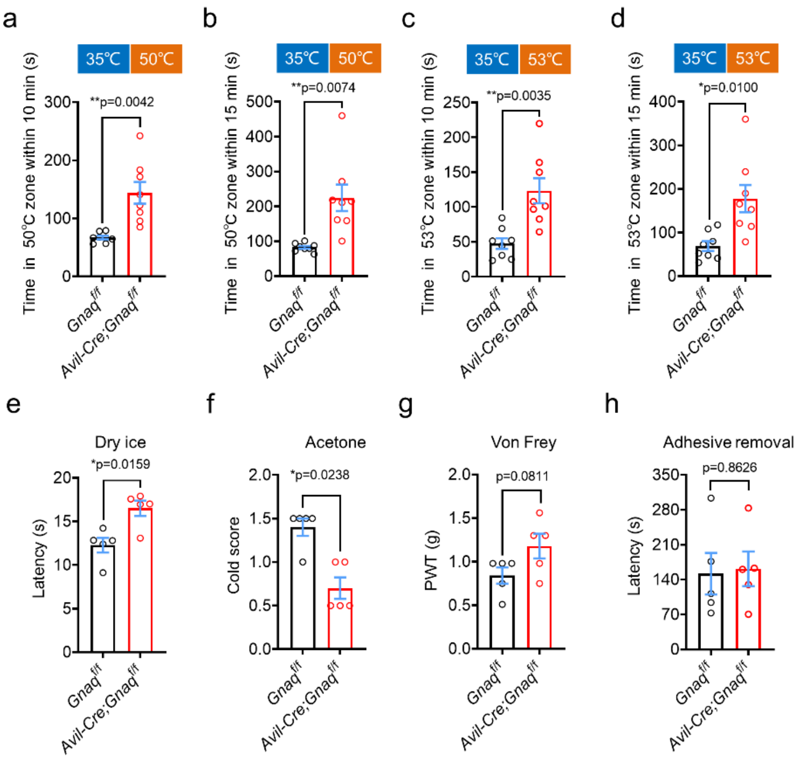

Supplementary Fig. S13 Behavioral tests of *Avil-Cre;Gnaq$^{f/f}$* mice.

a, **b** Temperature preference test of *Avil-Cre;Gnaq$^{f/f}$* mice at 35°C/50°C, the time mice spent in testing zone within 10 min (**a**) and 15 min (**b**) were counted. Welch's t-test; and n=8 mice/group.

c, **d** Temperature preference test of of *Avil-Cre;Gnaq$^{f/f}$* mice at 35°C/53°C, the time mice spent in testing zone within 10 min (**c**) and 15 min (**d**) were counted. Welch's t-test; and n = 8 mice/group.

e Dry ice test of *Avil-Cre;Gnaq$^{f/f}$* mice for examining cold sensitivity. Mann-Whitney U test. n = 5 mice/group.

f Acetone test of *Avil-Cre;Gnaq$^{f/f}$* mice for examining evaporative cooling sensitivity. Mann-Whitney U test. n = 5 mice/group.

g Von Frey test of *Avil-Cre;Gnaq$^{f/f}$* mice for examining mechanical sensitivity. Unpaired *t*-test. n = 5 mice/group.

h Adhesive removal test of *Avil-Cre;Gnaq$^{f/f}$* mice for examining touch sensitivity. Unpaired t-test. n = 5 mice/group.

All data are expressed as mean ±s.e.m. $*p < 0.05$, and $**p < 0.01$.

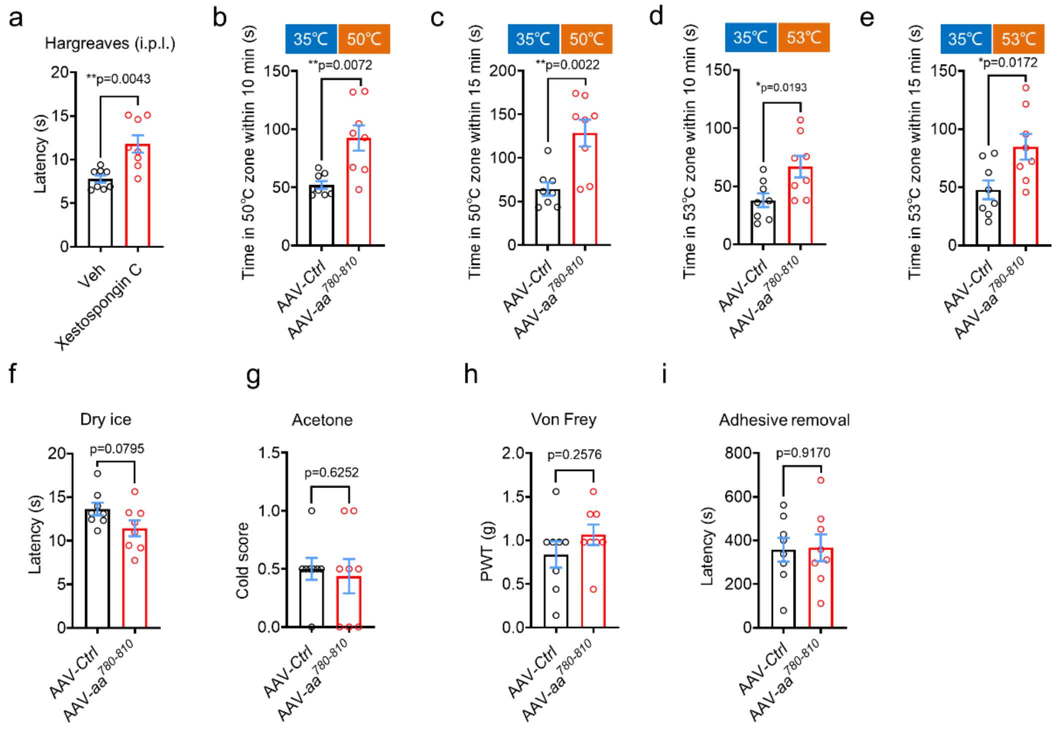

Supplementary Fig. S14 TMC6/Gαq signaling specifically mediates noxious heat sensation.

a Hargreaves test with 35% irradiation intensity of mice intraplantarly injected with Xestospongin C, 100 μM, 10 μl/mice. Welch's t-test. n = 8 mice/group.

b-i Behavioral tests of mice 8-weeks after i.p. injected with AAV2/9-*Ctrl* or AAV2/9-$aa^{780\text{-}810}$ at P0. n=8 mice/group. **b**, **c** Temperature preference test at 35°C/50°C, the time mice spent in testing area within 10 min (**b**) and 15 min (**c**) were counted. (**b**): Welch's t-test; (**c**): Unpaired t-test **d**, **e** Temperature preference test at 35°C/53°C, the time for mice spent in testing area within 10 min (**d**) and 15 min (**e**) were counted. Unpaired t-test. **f** Dry ice test for cold sensitivity. Unpaired t-test. **g** Acetone test for evaporative cooling sensitivity. Mann-Whitney U test. **h** Von Frey test for mechanical sensitivity. Unpaired t-test. **i** Adhesive removal test for touch sensitivity. Unpaired t-test.

All data are expressed as mean ± s.e.m. *p < 0.05, and **p < 0.01.

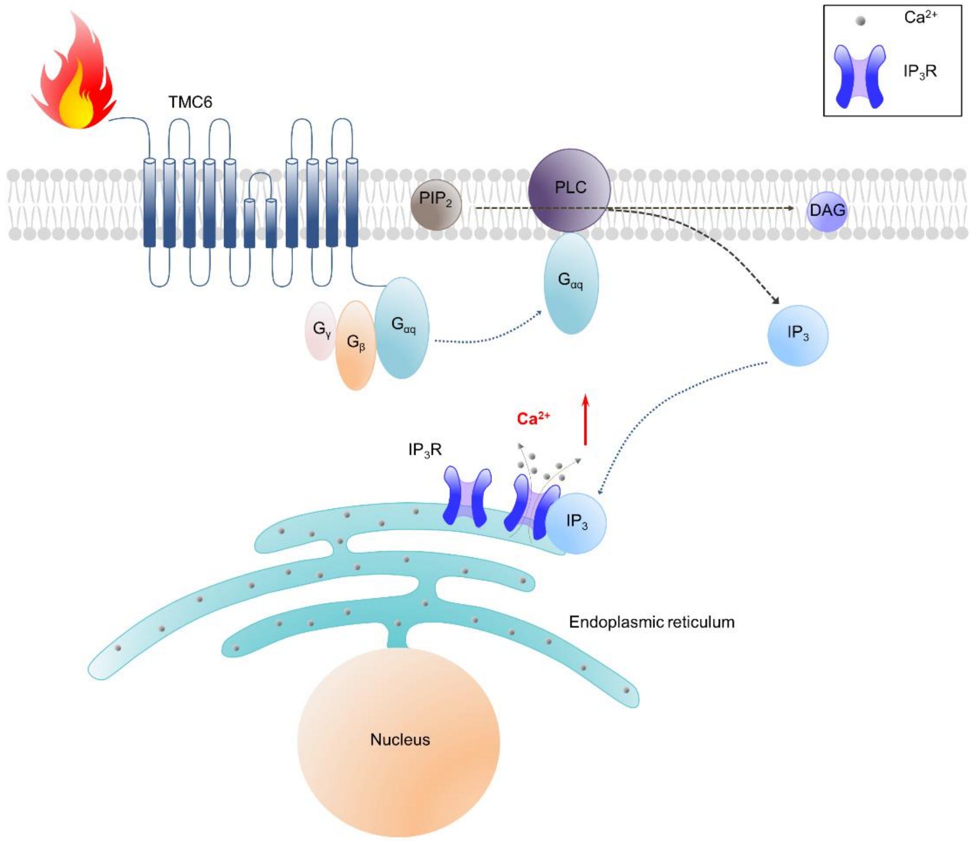

Supplementary Fig. S15 TMC6 mediates noxious heat sensation via Gαq signaling. Schematic diagram shows the process of TMC6 sensing noxious heat. In response to noxious heat, Gαq dissociates from TMC6 and activates phospholipase C (PLC), which hydrolyzes phosphatidylinositol 4, 5-diphosphate (PIP2) into diacylglycerol (DAG) and inositol triphosphate (IP3). IP3 acts on 1,4,5-trisphosphate receptors (IP3Rs) on ER, leading to the stored Ca^{2+} releasing from ER into cytosol and noxious heat transduction.

Supplementary methods and materials

In situ hybridization (ISH) and immunohistochemistry (IHC)

Mice were deeply anesthetized with 0.75% pentasorbital sodium (10 µl/g) and perfused from atrium sinistrum with 1 x PBS followed by 4% PFA, tissues were collected and postfixed in 4% PFA overnight and dehydrated in 30% sucrose for 2 days. Spinal cord (30 µm, free-floating), Brain (30 µm, free-floating), skin (14 µm, mounted section), and DRG (14 µm, mounted section) were cut in a cryostat (Leica). RNAscope™ Fluorescent Multiplex Assay kit and specific probes targeting mouse *Tmc6* (ACD, Cat No. 421001), *Gnaq* (ACD, Cat No. 1067091), and *Ip3r1* (ACD, Cat No. 488291) were used for ISH exactly following the protocol recommended by ACDs. For IHC, samples were incubated with primary antibodies: Anti-NF200 (mouse, 1:1000, Millipore; Cat No. AB_477257), anti-CGRP (goat, 1:1000; Bio-Rad; Cat No. AB_2290729), anti-TH (rabbit, 1:1000, Millipore; Cat No. AB152), Anti-GS (rabbit,1:1000,Abcam; Ca No. ab49873) Anti-NeuN (mouse,1:1000, Millipore; Cat No. MAB377) and anti-PGP 9.5 (rabbit, 1:2000, Proteintech; Cat No. 14730-1-AP) antibodies, followed by Cy3-(1:400, Jackson ImmunoResearch Laboratories Inc. Cat No.112-165-003),Cy5-(1:400, Jackson ImmunoResearch Laboratories Inc. Cat No.715-175-151), or FITC-conjugated secondary antibodies (1:400; Jackson ImmunoResearch Laboratories Inc. Cat No.115-095-003) or FITC-conjugated IB4 (10 µg/ml; Invitrogen. Cat No. I21411). Sections were examined under a Nikon fluorescence microscope and Leica SP5 confocal microscope.

Genotyping

For the purification of genomic DNA, mouse ears were collected and digested in a solution containing 25 mM NaOH and 0.2 mM EDTA at 95°C for one hour. This was followed by neutralization with 40 mM Tris-HCl (pH 5.5). *Tmc6* knockout mice were genotyped using primers 5'-ttgttacaagagtggattgggtgg-3', 5'-taaagaggctcaggccatacatc-3', and 5'-ggtattagagaggaggaaggggac-3' with wildtype band size 450 bp, and knockout band size approximately 500 bp. The primers for genotyping *Advillin-cre* mice were 5'-tgccacgaccaagtgacagcaatg-3' and 5'-accagagacggaaatccatcgctc-3' with band size of about 400 bp. The primers for genotyping *Gnaq* flox mice were 5'-

ggtttttctttgaggcagtcatgc-3' and 5'-atcagactcaggtcctcatgactgcaa-3' with wildtype band size 277 bp and *Gnaq* flox band size 382 bp separately. The primer for genotyping *Tmc6* flox mice were 5'-ccatgaggaggcaattctgacc-3' and 5'-aacataaagactttcaggggcca-3' with wildtype band size 136 bp and *Tmc6* flox band size 210 bp separately.

Behavioral tests

Evaporative cooling test: mice were habituated in a box placed on an elevated metal mesh floor for 30 min. 30 μl acetone was applied to the bottom of a hind paw using a pipette. Responses to acetone were divided into four levels: 0, no response; 1, quick withdrawal, flick or stamp the paw; 2, prolonged withdrawal or repeatedly flick; 3, repeatedly flick with licking ventral side of the paw.

Dry ice test: mice were habituated in a box placed on an elevated glass floor for 30 min. Cylindrical dry ice was applied directly to the glass opposite to the bottom of the hand paw. The average withdrawal latency of both hind paws was recorded as the latency of dry ice. The cutoff value is 20 s.

Adhesive removal test: mice were habituated in a box placed on an elevated glass floor for 15 min one day before test. Each hind paw of mice was stuck with an adhesive round label (diameter 6 mm). Animal performance was recorded by video camera for 15 min, the time needed to sense the adhesive labels was calculated as the latency of adhesive removal test.

Hot plate test: BIOSEB cold-hot-plate was used to test the mice's response to heat. The duration since putting mice on the plate to the mice showing licking or flicking behavior was recorded as latency of hot plate test. Mice were tested at 48°C, 50°C, 53°C, and 56°C, and cut-off times were set as 60 s, 50 s, 30 s, and 20 s respectively.

Tail flick test:

Tail flick test: For tail flick test, mice were loosely wrapped in a rodent restrainer, and their tails were immersed about 2 cm into water heated to 45°C. The time after immersion at which the tail flick response was noted was recorded. For 45°C, cutoff time for this test was 120 s.

Hargreaves test: mice were habituated in a testing box placed on an elevated glass pane for 30 min. Hargreaves Apparatus (Ugo Basile) was used to stimulate the left hind

paw of mice with 35% irradiation intensity. The time since turning on the light to mice showing flicking behavior to avoid light stimulus was recorded as latency. Cutoff was set as 25 s.

Von Frey test: to test mechanical sensitivity, we confined mice in testing boxes placed on an elevated metal mesh floor, and stimulated their hind paws using a series of von Frey hairs with increasing stiffness (0.16–2.00 g; North Coast Medical), perpendicularly to the central plantar surface. We determined the 50 % paw withdrawal threshold by Dixon's up-down method [51].

Temperature preference test: in the temperature preference test, the KW-RTD Thermal Gradient Instrument (KEW BASIS) was used, where each zone can be set to any temperature within the range of 4°C to 56°C. Before the test, mice were allowed to freely explore two adjacent zones (each measuring 14 cm × 14 cm × 10 cm) for 15 minutes at room temperature. Afterwards, the temperature was rapidly adjusted to the predetermined setting. The activity of the mice was then recorded for another 15 minutes using a camera. The time mice spent in the test area within 5, 10, and 15 minutes was calculated using Anymaze software.

Open field: mice were put in a box (40 cm x 40 cm) and their locomotor activities were recorded for 10 min. The data were analyzed by JLBehv-LAG-4 system. Total distance and average velocity were measured for the evaluation of the motor function.

Rotarod: mice were pre-trained on the rotarod (med associates inc) twice a day in 5 rpm for 2 days. On the third day, mice were tested on a rotarod with the velocity increasing from 4 rpm to 34 rpm within 300s. The durations on the rotarod before the mice fell off were recorded by the Rota-Rod 2 software (med associates inc).

RT-PCR and qRT-PCR

Tissues or cells were rapidly isolated in RNase-free condition. Total RNAs were extracted with Trizol. RNAs (0.5-1 μg) were reverse-transcribed using with 5xAll in one RT MasterMix (abmart). RT-PCR was used to test the mRNA level of *Tmc6* in DRG of WT mice and *Tmc6*$^{-/-}$ mice with primer: Forward primer: 5'-cacacacacagttat-3', Reverse primer: 5'-gatgcaggtaaggaag-3'. qRT-PCR was performed using Power SYBR Green to analyze the expression levels of *Tmc*1-8 in mouse DRG with primers listed in

Supplemental Table 2. To test the mRNA levels of *Tmc6* in HEK293T cells and human DRG, we used the primers listed in Supplemental Table 2.

Plasmid construction

The cDNA of TMC6 was cloned from mouse DRG into the backbone of pCAG-IRES-*egfp*, and subcloned into p*egfp*-N3 fused with 2xHA tag at NH2-terminus. p*egfp*-N3-2HA-m*Tmc6* was used to generate mutants, including p*egfp*-N3-2HA-m*Tmc6*$^{\Delta 740\text{-}759}$, p*egfp*-N3-2HA-m*Tmc6*$^{\Delta 760\text{-}779}$, p*egfp*-N3-2HA-m*Tmc6*$^{\Delta 780\text{-}799}$, p*egfp*-N3-2HA-m*Tmc6*$^{\Delta 800\text{-}810}$ and p*egfp*-N3-2HA-m*Tmc6*$^{\Delta 780\text{-}810}$. The cDNA of mouse Gαq (*Gnaq*) was cloned from mouse DRG into pCAG-IRES-*egfp* tagged with 3xFlag at the COOH-terminus. KOD-Plus-Neo (TOYOBO) and Q5 Site-Directed Mutagenesis Kit (NEB, E0554S) were used to clone and generate mutants. All primers and oligonucleotides for the construction of plasmids are listed in Supplementary Table S2.

Cell culture and transfection

CHO cells were cultured in F12K media supplemented with 10% FBS (heat-inactivated) and 1% Penicillin-Streptomycin Solution (PS) at 37°C under 5% CO_2. ND7/23 cells were cultured in DMEM media (none L-Glutamine) supplemented with 10% FBS, 1% L-Glutamine, and 1% PS at 37°C under 5% CO_2. HEK293T and HEK293 cells were cultured in DMEM media mixed with 10% FBS and 1% PS at 37°C under 5% CO_2. For primary DRG neuron culture, DRG were dissected from 3-8 week-old mice and digested with collagenase (1 mg/ml, Sigma-Aldrich)/Trypsin (0.4 mg/ml, Sigma-Aldrich)/Deoxyribonuclease I (0.1 mg/ml) for 45 min. Cells were placed on glass coverslips coated with poly-D-lysine and grown in a neurobasal defined medium (10% fetal bovine serum, 2% B27 and 1% PS supplement) at 37°C with 5% CO_2 for 24 h before experiments. Plasmids were transfected into CHO cells, ND7/23 cells, HEK293 cells or HEK293T cells with Lipofectamine™ 3000 (ThermoFisher) following suggested procedure. For electroporation of DRG neurons, Cells were placed within an electroporation buffer supplemented with 6 μg of pCAG-IRES-*egfp*-m*Tmc6* or pCAG-IRES-*egfp* plasmid and then electroporated using the Nucleofector II (Amaxa) with the O-003 program. The cells were harvested 48 hr after transfection for further analysis.

Cell surface biotinylation

Transfected CHO cells were rinsed with ice-cold PBS containing Ca^{2+} and Mg^{2+}, and subsequently treated with EZ-Link Sulfo-NHS-LC-Biotin (Thermo Scientific™, 21217) on a shaker at 4°C for 60 minutes. The biotinylation reaction was terminated using glycine, followed by cell lysis using RIPA Buffer. The resultant lysates were incubated with Streptavidin Agarose Resin (Thermo Scientific™, 20353) overnight at 4°C on a rotor, a step critical for the isolation of plasma membrane proteins. This was followed by incubating the beads in 1 x SDS-PAGE loading buffer at 50°C for 20 minutes. Subsequent analysis of the samples was performed via Western blot, employing Transferrin Receptor (TfR) as an internal control. Image J software facilitated the quantification of the grayscale values in the Western blot results. The cell surface localization of TMC6 was quantified by calculating the intensity of the HA-tagged protein bands in both the surface-bound and lysate fractions, followed by a two-step normalization process.

Nonpermeable live cell labeling and immunocytochemistry

Transfected CHO cells were cultured on Poly-D-Lysine coated coverslips for 24-48 hr at 37°C under 5% CO_2. Cells were washed with ice-cold Ca^{2+}-Mg^{2+}-PBS for three times and incubated with anti-HA primary antibody (mouse, 1:100, abmart; M2003M) at 4°C for 1 hr. Later, cells were incubated with Cy3-conjugated secondary antibody (1:400; Jackson ImmunoResearch Laboratories Inc,Cat No.112-165-003) at 4°C for 30 min and examined under Olympus FV3000 confocal laser scanning microscope.

Calcium imaging

Transfected HEK293T cells or primarily cultured DRG neurons were stained with fura-2 (5 μM) and F-127 (5 μM) at 37°C for 30 min followed by 3 times washing with extracellular solution (130 mM NaCl, 5 mM KCl, 2 mM KH_2PO_4, 2.5 mM $CaCl_2$, 1 mM $MgCl_2$, 10 mM Hepes, 10 mM Glucose, pH 7.2 and osmolarity 295-300 mOsm). For detecting internal Ca^{2+} release, DRG neurons were preincubated in Ca^{2+}-free bath solution (normal extracellular solution free from $CaCl_2$ containing 10 mM EGTA) with 2.5 μM TG for 15 min, 10 μM U73122 for 15 min, 20 μM 2-APB for 10 min, 10 μM Ryanodine for 20 min, 3 μM FCCP for 5 min and 100 nM bafilomycin A1 for 15 min. Heat stimulation was also given to DRG neurons in Ca^{2+}-free extracellular solution

(130 mM NaCl, 5 mM KCl, 2 mM KH_2PO_4, 1 mM $MgCl_2$, 10 mM Hepes, 10 mM Glucose, 10 mM EGTA, pH 7.2 and osmolarity 295-300 mOsm).

Initially, the temperature of extracellular solution was set at 37°C. Before starting the recording, adjust the parameters of the 340 and 380 channels to make the intensity ratio of 340 and 380 as close to 1 as possible. VisiView Image acquisition Software with Ratio Option system (Visitron system) was used to acquire fluorescence intensity at 340 nm and 380 nm fluorescence separately. Once the recording begins, first record a baseline for 100 seconds, then gradually increase the temperature to either 42°C or 45°C over approximately 100 seconds, maintain this temperature for about 200 seconds, and then start cooling to 37°C. Around 700 seconds, administer a 50mM KCl stimulus, followed by elution, and conclude the recording around 1000 seconds. In the heat stimulation process, a Warner TC344C Temperature Controller was employed to progressively increase the water bath temperature from 37°C to 42°C (or 45°C) over a duration of 100 seconds. Simultaneously, a BCL-X multipath temperature recorder was utilized for the continuous monitoring and recording of the water bath's temperature throughout the experiment. During the calcium signaling recording process, we utilized a Nikon Plan Fluor 10x/0.3 WD 3.5 objective, notable for its 200 mm focal length. To address potential focus shifts caused by minor thermal drift during the heating process, we employed manual focusing as previously reported[10].

The statistical indicators include ΔR/R0, response rate, and spike number. ΔR/R0 is defined as (Rmax − R0)/R0, where R0 is the initial Fura2 ratio (340/380) averaged over the first 100 seconds, and Rmax is the maximal Fura2 ratio (340/380) observed during heat stimulation. Response rate refers to the percentage of responsive DRG neurons during heat stimulation, defined as those with ΔR/R0 >5 times of s.e.m in the presence of extracellular Ca^{2+}. Spike number refers to the number of calcium peaks or spikes that occur during the process of thermal stimulation. In the absence of external calcium, the calcium signaling changes induced by heat stimulation are relatively small. To more convincingly demonstrate the intergroup differences, we have presented the data from all cells. The specific method is as follows: the F0 level of each cell is normalized to 1, and the ratio value of the cell after 100 seconds is also normalized at

the same rate. The normalized curves of all cells are fitted, and presented as mean ± s.e.m.

Whole-cell patch clamp recording

Whole-cell recordings were conducted at room temperature using an Axon-700B amplifier with a Digidata-1550B (Axon Instruments, Sunnyvale, CA). For sodium currents recording, small DRG neurons cultured within 24 hours were used for voltage-clamp recordings. The pipettes were pulled from borosilicate glass with a tip resistance of 5 to 7 MΩ. The pipette solution for recording sodium current contained (in mM): 140 CsF, 10 NaCl, 10 HEPES, 1 EGTA, PH7. 4 with CsOH (310mOsmol/L adjusted with sucrose). The extracellular solution for cultured DRG neuron recording contained (in mM): 140 NaCl, 3 KCl, 20 TEACl, 1 CaCl2, 1 MgCl2, 0.1 CdCl2, 3 4-aminopyridine, 10 HEPES, 10 glucose, adjusted to pH 7.3 with NaOH. (320mOsmol/L adjusted with sucrose). Membrane currents were filtered at 2 kHz and digitized at 10 kHz.

Under voltage-clamp recording mode, cells were clamped at -60mV followed by a test pulse ranging from -80 to +60mV in increments of 10 mV in the absence of TTX to evoke total sodium current. The maximal peak current at various voltages was used for current density analysis.

Current-clamp recording

Primarily cultured DRG neurons were placed in a temperature-controlled chamber of around 2 ml volume of extracellular solution at around 30°C. The extracellular solution contains the following (in mM): 130 NaCl, 5 KCl, 2 KH2PO4, 2.5 CaCl2,1MgCl2,10 HEPES and 10 glucose, pH 7.2 (295–300 mOsm). Pipettes (3–5 MΩ) were filled with solution contained (in mM): 140 CsCl, 10 EGTA, 10 HEPES and 2Mg–ATP, adjusted to pH 7.3 with CsOH. Temperature control was performed by Warner TC-344C Temperature Controller.

Neurons were clamped at -60mV in voltage-clamp mode and then the recording was switched to current-clamp mode. In current-clamp mode, neurons were adjusted to a membrane potential of about -50mV by injection of a DC current. The temperature ranged from 30°C to 45°C in about 120s. The heat-sensitive neuron was considered if

the AP firing could be evoked by the increasing temperature in the small neuron. In order to test whether the internal Ca^{2+} release is involved in noxious heat-induced Aps, we incubated DRG neurons from C57BL/6J mice with 5 μM Xestospongin C for 1h. The analysis of each heat-sensitive neuron including the AP distribution and amplitudes within the temperature ranging from 38°C to 45°C, the change of AP amplitude in noxious heat was quantified by the ratio of the AP amplitude at 45°C to that of the first AP.

Experimental current-clamp data were analyzed using Clampfit (version 10.7.0.3; Molecular Devices, LLC.), Matlab (R2022b Update 4; The MathWorks, Inc.), and Microsoft Excel software programs.

Co-immunoprecipitation (Co-IP)

Transfected ND7/23 cells were washed three times with ice-cold PBS and lysed with RIPA Buffer (10 mM Tris-HCl, pH 7.4, 150 mM NaCl, 1 mM EDTA, 1% Triton X-100 and 10% glycerol). The lysate was centrifugated at 13,000 rpm for 15 min to remove the debris. Then, 40 μl supernatant was collected as whole-cell lysate, and the remaining supernatant was precipitated with 2 μg antibody at 4°C overnight followed by protein A/G beads incubation (Santa Cruz Bicycles) at 4°C for 2 hr. Immunoprecipitates were collected and washed at least three times with the immunoprecipitation buffer. The whole-cell lysate or beads were then incubated at 50°C for 30 min in SDS-PAGE loading buffer for Western blot analysis.

Western blot

The samples were separated on an SDS-PAGE gel, transferred, and probed with antibodies against HA tag (mouse, 1:5000, abmart; M2003M or rabbit, 1:5000, Cell Signaling Technology; 3724), Flag tag (mouse, 1:5000, abmart; M2008M or rabbit, 1:5000, Cell Signaling Technology;14793), or transferrin receptor (mouse, 1:2000, Life technology; AB_2533029). The immunoreactive bands were then detected with corresponding horseradish peroxidase-conjugated secondary antibodies (mouse, 1:5000, Jackson ImmunoResearch Labs; AB_10015289 and rabbit, 1:5000, Jackson ImmunoResearch Labs, AB_2313567), visualized with enhanced chemiluminescence (Beyotime) and quantified with Image J software (National Institutes of Health). Each

experiment was repeated at least three times.

Immunoprecipitation-Mass Spectrometry analysis (IP-MS)

Transfected ND7/23 cells were lysed with ice-cold RIPA and centrifugated at 13,000 rpm for 15 min. The supernatant was collected and equally split into two parts. The same amount of control IgG and anti-HA antibody were added into these two parts of supernatant separately and incubated a 4°C overnight followed by protein A/G beads incubation (Santa Cruz Bicycles) at 4°C for 2 hr. 1x Loading Buffer (Beyotime, 60 μl) were added into beads heated at 50°C for 20 min. Then samples were separated on an SDS-PAGE gel and subjected to Mass spectrometry analysis.

Firstly, the gel was cut into small pieces, gel-included proteins were reduced in 10 mM DTT at 56 °C for 1 h, and then alkylated in the dark with 55 mM iodoacetamide at room temperature for 45 min. The samples were washed with 25mM NH_4HCO_3, 50mM NH_4HCO_3/acetonitrile 1:1, acetonitrile once each, and dehydrated by acetonitrile until the colloidal granules turned white. After that, the Trypsin (0.1 μg/μl Promega, Madison) was diluted with 25 mM NH_4HCO_3 and the proteins were digested at 4°C for 30min at an enzyme/substrate mass ratio of 1:50. After the solution was fully absorbed by the samples, excess enzyme liquid was moved and the samples were digested in 25mM NH_4HCO_3 overnight at 37°C. After digestion, samples were acidified with 1% formic acid and the liquid phase was retained after centrifugation. The solid phase was extracted with 60% acetonitrile 0.1% formic acid twice for the liquid phase and the solution extracted three times were combined. The solution was vacuum centrifugal dried and 0.1% formic acid was used to dissolve peptides. Then LC-MS/MS analysis of fraction samples was performed on a nano-HPLC chromatography system coupled to a hybrid trapped ion mobility spectrometry quadrupole time-of-flight mass spectrometer (TIMS-TOF Pro, Bruker Daltonics, Billerica, MA). The MS data were then searched against the Swiss-Prot database (downloaded on August 20, 2020, containing 20375 protein sequence entries) using PEAKS Online Xpro Software (v1.6) to identify the peptide and protein. Mass tolerances were set as 15 ppm for precursor ions and 0.05 Da for fragments. Carbamidomethyl on C was set as fixed modification while Oxidation on M and Acetyl at protein N-terminus were set as variable

modifications. False discovery rate (FDR) at PSM and protein level was controlled below 1%.

The change rate of exponentially modified protein abundance index (emPAI) was calcμlated for each protein that appeared in the control group as follows.

$$R_{\Delta emPAIe} = \frac{emPAIe - emPAIc}{emPAIc}$$

($R_{\Delta emPAI}$: emPAI change rate; *emPAIe*: emPAI of the protein in the experimental group; *emPAIc*: emPAI of the protein in the control group).

The $R_{\Delta emPAI}$ of the proteins that only appeared in the experimental group was set to be a number bigger than any other $R_{\Delta emPAI}$ instead of being calculated because their *emPAIc* equals to 0. All the proteins were identified by their accession names and the corresponding descriptions shown in the MS result report. Then the proteins were sorted by *emPAIc* in descending order for selection and were represented as log (Score).

Supplementary Tables

Supplementary Table S1 Immunoprecipitation-Mass Spectrometry (IP-MS) analysis of proteins interacts with TMC6 in ND7/23 cells.

Immunoprecipitation-Mass Spectrometry (IP-MS) analysis of proteins interacts with TMC6 in ND7/23 cells									
	Protein		Description	core-contr	re-experim	PAI-cont	AI-experin	re-differe	PAI-differe
Chaperon	HSP7C_MOUSE	1	Heat shock cognate 71 kDa protein OS=Mus musculus OX=10090 GN=Hspa8 PE=1 SV=1	0	1346	0	1.66	999999	999999
	A0A0G2K793_RAT	1	Heat shock protein HSP 90-beta OS=Rattus norvegicus OX=10116 GN=Hsp90ab1 PE=1 SV=1	0	280	0	0.21	999999	999999
	A0A0G2JTG1_RAT	1	10 kDa heat shock protein, mitochondrial OS=Rattus norvegicus OX=10116 GN=Hspe1 PE=1 SV=	0	51	0	0.82	999999	999999
	DNJA1_MOUSE	1	DnaJ homolog subfamily A member 1 OS=Mus musculus OX=10090 GN=Dnaja1 PE=1 SV=1	0	46	0	0.07	999999	999999
	TCPH_MOUSE	1	T-complex protein 1 subunit eta OS=Mus musculus OX=10090 GN=Cct7 PE=1 SV=1	0	37	0	0.05	999999	999999
	STIP1_MOUSE	1	Stress-induced-phosphoprotein 1 OS=Mus musculus OX=10090 GN=Stip1 PE=1 SV=1	0	32	0	0.05	999999	999999
	CALX_MOUSE	1	Calnexin OS=Mus musculus OX=10090 GN=Canx PE=1 SV=1	0	31	0	0.05	999999	999999
Membran	TMC6_MOUSE	2	Transmembrane channel-like protein 6 OS=Mus musculus OX=10090 GN=Tmc6 PE=1 SV=2	0	614	0	0.26	999999	999999
	ANXA2_MOUSE	2	Annexin A2 OS=Mus musculus OX=10090 GN=Anxa2 PE=1 SV=2	0	84	0	0.17	999999	999999
	ENV1_MOUSE	2	MLV-related proviral Env polyprotein OS=Mus musculus OX=10090 PE=1 SV=3	0	70	0	0.04	999999	999999
	A0A0H2UHA6_RAT	2	Transmembrane protein 33 OS=Rattus norvegicus OX=10116 GN=Tmem33 PE=1 SV=1	0	33	0	0.11	999999	999999
	F1LS74_RAT	2	Slit homolog 1 protein OS=Rattus norvegicus OX=10116 GN=Slit1 PE=4 SV=2	0	30	0	0.02	999999	999999
	A0A0G2JVW2_RAT	2	Voltage-dependent R-type calcium channel subunit alpha OS=Rattus norvegicus OX=10116 GN=C	0	21	0	0.01	999999	999999
	PALM_MOUSE	2	Paralemmin-1 OS=Mus musculus OX=10090 GN=Palm PE=1 SV=1	0	19	0	0.07	999999	999999
	F1LRH9_RAT	2	Transient receptor potential cation channel subfamily A member 1 OS=Rattus norvegicus OX=101	0	15	0	0.02	999999	999999
	A0A0G2JXN8_RAT	2	Oxysterol-binding protein OS=Rattus norvegicus OX=10116 GN=Osbpl8 PE=1 SV=1	0	45	0	0.03	999999	999999
GTPase	DOCK4_MOUSE	3	Dedicator of cytokinesis protein 4 OS=Mus musculus OX=10090 GN=Dock4 PE=1 SV=1	0	610	0	0.29	999999	999999
	ELMO2_MOUSE	3	Engulfment and cell motility protein 2 OS=Mus musculus OX=10090 GN=Elmo2 PE=1 SV=1	0	557	0	0.72	999999	999999
	B0BMW4_RAT	3	GNAS complex locus OS=Rattus norvegicus OX=10116 GN=Gnas PE=2 SV=1	0	95	0	0.22	999999	999999
	G3BP1_MOUSE	3	Ras GTPase-activating protein-binding protein 1 OS=Mus musculus OX=10090 GN=G3bp1 PE=1	0	62	0	0.06	999999	999999
	GNAQ_MOUSE	3	Guanine nucleotide-binding protein G(q) subunit alpha OS=Mus musculus OX=10090 GN=Gnaq PE	0	48	0	0.07	999999	999999
	RALA_MOUSE	3	Ras-related protein Ral-A OS=Mus musculus OX=10090 GN=Rala PE=1 SV=1	0	36	0	0.13	999999	999999
	PLCE1_RAT	3	1-phosphatidylinositol 4,5-bisphosphate phosphodiesterase epsilon-1 OS=Rattus norvegicus OX=1	0	19	0	0.01	999999	999999
	A0A0G2JVF0_RAT	3	Rho GTPase activating protein 4, isoform CRA_a OS=Rattus norvegicus OX=10116 GN=Arhgap4	0	15	0	0.03	999999	999999
	ZO2_MOUSE	3	Tight junction protein ZO-2 OS=Mus musculus OX=10090 GN=Tjp2 PE=1 SV=2	0	60	0	0.02	999999	999999
Enzyme	PP1G_MOUSE	5	Serine/threonine-protein phosphatase PP1-gamma catalytic subunit OS=Mus musculus OX=1009(	0	92	0	0.08	999999	999999
	B1H262_RAT	5	Protein phosphatase 1, regulatory subunit 9B OS=Rattus norvegicus OX=10116 GN=Ppp1r9b PE=	0	82	0	0.07	999999	999999
	RACK1_MOUSE	5	Receptor of activated protein C kinase 1 OS=Mus musculus OX=10090 GN=Rack1 PE=1 SV=3	0	66	0	0.09	999999	999999
	PP1A_MOUSE	5	Serine/threonine-protein phosphatase PP1-alpha catalytic subunit OS=Mus musculus OX=10090 (	0	60	0	0.08	999999	999999
	ASM3B_MOUSE	5	Acid sphingomyelinase-like phosphodiesterase 3b OS=Mus musculus OX=10090 GN=Smpdl3b PE	0	49	0	0.06	999999	999999
	CDK3_MOUSE	5	Cyclin-dependent kinase 3 OS=Mus musculus OX=10090 GN=Cdk3 PE=1 SV=2	0	48	0	0.19	999999	999999
	SRC8_MOUSE	5	Src substrate cortactin OS=Mus musculus OX=10090 GN=Cttn PE=1 SV=2	0	48	0	0.05	999999	999999
	PSA1_MOUSE	5	Proteasome subunit alpha type-1 OS=Mus musculus OX=10090 GN=Psma1 PE=1 SV=1	0	47	0	0.11	999999	999999
	ADRM1_MOUSE	5	Proteasomal ubiquitin receptor ADRM1 OS=Mus musculus OX=10090 GN=Adrm1 PE=1 SV=2	0	39	0	0.07	999999	999999
	A0A0G2K0W9_RAT	5	Proteasome subunit alpha type OS=Rattus norvegicus OX=10116 GN=Psma7 PE=1 SV=1	0	38	0	0.11	999999	999999
	A0A0G2JSL0_RAT	5	Proteasome subunit beta OS=Rattus norvegicus OX=10116 GN=LOC100360846 PE=3 SV=1	0	34	0	0.12	999999	999999
	A0A0G2K0X8_RAT	5	Alkaline phosphatase OS=Rattus norvegicus OX=10116 GN=Alpl PE=1 SV=1	0	33	0	0.05	999999	999999
	PSA4_MOUSE	5	Proteasome subunit alpha type-4 OS=Mus musculus OX=10090 GN=Psma4 PE=1 SV=1	0	30	0	0.11	999999	999999
	D3ZVQ0_RAT	5	Ubiquitin carboxyl-terminal hydrolase OS=Rattus norvegicus OX=10116 GN=Usp5 PE=1 SV=1	0	28	0	0.03	999999	999999
	A0A0G2JUM4_RAT	5	1-acylglycerol-3-phosphate O-acyltransferase 1 (Fragment) OS=Rattus norvegicus OX=10116 GN	0	26	0	0.16	999999	999999
	DI3L1_MOUSE	5	DIS3-like exonuclease 1 OS=Mus musculus OX=10090 GN=Dis3l PE=1 SV=2	0	23	0	0.03	999999	999999
	LDHB_MOUSE	5	L-lactate dehydrogenase B chain OS=Mus musculus OX=10090 GN=Ldhb PE=1 SV=2	0	22	0	0.18	999999	999999
	D4AEH3_RAT	5	Proteasome (Prosome, macropain) 26S subunit, non-ATPase, 7 (Predicted) OS=Rattus norvegicu	0	20	0	0.18	999999	999999
	D4AEH9_RAT	5	4-alpha-glucanotransferase OS=Rattus norvegicus OX=10116 GN=Agl PE=1 SV=1	0	20	0	0.02	999999	999999
	ARBK1_MOUSE	5	Beta-adrenergic receptor kinase 1 OS=Mus musculus OX=10090 GN=Grk2 PE=1 SV=2	0	17	0	0.04	999999	999999
	A0A0G2K1A0_RAT	5	N-acetyltransferase domain-containing protein OS=Rattus norvegicus OX=10116 GN=LOC102553	0	17	0	0.16	999999	999999
	A0A0G2K858_RAT	5	E3 ubiquitin-protein ligase RNF8 OS=Rattus norvegicus OX=10116 GN=Rnf8 PE=3 SV=1	0	17	0	0.06	999999	999999
	F1LWT0_RAT	5	SUMO-interacting motifs-containing 1 OS=Rattus norvegicus OX=10116 GN=Simc1 PE=4 SV=3	0	16	0	0.02	999999	999999
	ARGI1_MOUSE	5	Arginase-1 OS=Mus musculus OX=10090 GN=Arg1 PE=1 SV=1	0	16	0	0.09	999999	999999
	A0A0G2K400_RAT	5	Janus kinase and microtubule-interacting protein 3 OS=Rattus norvegicus OX=10116 GN=Jakmip3	0	16	0	0.03	999999	999999
	PPCS_MOUSE	5	Phosphopantothenate--cysteine ligase OS=Mus musculus OX=10090 GN=Ppcs PE=1 SV=1	0	15	0	0.09	999999	999999
	CNDP1_RAT	5	Beta-Ala-His dipeptidase OS=Rattus norvegicus OX=10116 GN=Cndp1 PE=1 SV=1	0	15	0	0.06	999999	999999
	BCAT1_MOUSE	5	Branched-chain-amino-acid aminotransferase, cytosolic OS=Mus musculus OX=10090 GN=Bcat1	0	14	0	0.07	999999	999999
Other	A0A0G2K099_RAT	4	ATP synthase F1 subunit alpha OS=Rattus norvegicus OX=10116 PE=1 SV=1	0	164	0	0.32	999999	999999
	A0A0G2K8B7_RAT	4	RNA helicase OS=Rattus norvegicus OX=10116 GN=Eif4a2 PE=1 SV=1	0	128	0	0.16	999999	999999
	NUCL_MOUSE	4	Nucleolin OS=Mus musculus OX=10090 GN=Ncl PE=1 SV=2	0	137	0	0.17	999999	999999
	B2RYA6_RAT	7	Papillary renal cell carcinoma (Translocation-associated) (Predicted) OS=Rattus norvegicus OX=1	0	137	0	0.19	999999	999999
	M0RCL5_RAT	4	Histone H2A OS=Rattus norvegicus OX=10116 GN=LOC100910554 PE=1 SV=1	0	116	0	0.51	999999	999999
	RL11_MOUSE	4	60S ribosomal protein L11 OS=Mus musculus OX=10090 GN=Rpl11 PE=1 SV=4	0	113	0	0.34	999999	999999
	D3ZAZ0_RAT	4	Eukaryotic translation initiation factor 3 subunit M OS=Rattus norvegicus OX=10116 GN=Eif3m PE	0	111	0	0.07	999999	999999
	ROA1_MOUSE	4	Heterogeneous nuclear ribonucleoprotein A1 OS=Mus musculus OX=10090 GN=Hnrnpa1 PE=1 S\	0	88	0	0.19	999999	999999
	A0A0G2JTG7_RAT	4	Heterogeneous nuclear ribonucleoprotein H OS=Rattus norvegicus OX=10116 GN=Hnrnph1 PE=1	0	82	0	0.13	999999	999999
	F1LTW8_RAT	4	40S ribosomal protein S23 OS=Rattus norvegicus OX=10116 PE=3 SV=2	0	82	0	0.2	999999	999999
	PSMD2_MOUSE	4	26S proteasome non-ATPase regulatory subunit 2 OS=Mus musculus OX=10090 GN=Psmd2 PE=	0	82	0	0.09	999999	999999
	IMB1_MOUSE	4	Importin subunit beta-1 OS=Mus musculus OX=10090 GN=Kpnb1 PE=1 SV=2	0	55	0	0.03	999999	999999
	H14_MOUSE	4	Histone H1.4 OS=Mus musculus OX=10090 GN=H1-4 PE=1 SV=2	0	54	0	0.14	999999	999999
	G3V7L6_RAT	4	26S proteasome AAA-ATPase subunit RPT1 OS=Rattus norvegicus OX=10116 GN=Psmc2 PE=1	0	53	0	0.06	999999	999999
	PRS6B_MOUSE	4	26S proteasome regulatory subunit 6B OS=Mus musculus OX=10090 GN=Psmc4 PE=1 SV=2	0	49	0	0.07	999999	999999
	A0A0H2UHH9_RAT	4	40S ribosomal protein S24 OS=Rattus norvegicus OX=10116 GN=Rps24 PE=3 SV=1	0	48	0	0.21	999999	999999
	A0A096MKH2_RAT	7	Similar to RIKEN cDNA 1110059P08 OS=Rattus norvegicus OX=10116 GN=Vta1 PE=1 SV=1	0	48	0	0.09	999999	999999
	D3ZSF2_RAT	4	Similar to 60S ribosomal protein L26 (Silica-induced gene 20 protein) (SIG-20) OS=Rattus norvegic	0	42	0	0.19	999999	999999
	YBOX1_MOUSE	4	Y-box-binding protein 1 OS=Mus musculus OX=10090 GN=Ybx1 PE=1 SV=3	0	41	0	0.09	999999	999999
	EFHD2_RAT	7	EF-hand domain-containing protein D2 OS=Rattus norvegicus OX=10116 GN=Efhd2 PE=1 SV=1	0	41	0	0.25	999999	999999
	A0A0G2K850_RAT	4	RNA-binding protein EWS-like OS=Rattus norvegicus OX=10116 GN=LOC100912481 PE=1 SV=1	0	38	0	0.05	999999	999999
	SYRC_MOUSE	4	Arginine--tRNA ligase, cytoplasmic OS=Mus musculus OX=10090 GN=Rars1 PE=1 SV=2	0	34	0	0.04	999999	999999
	CNTN3_MOUSE	6	Contactin-3 OS=Mus musculus OX=10090 GN=Cntn3 PE=1 SV=2	0	33	0	0.03	999999	999999
	A0A0U1RVI9_RAT	4	40S ribosomal protein S2 OS=Rattus norvegicus OX=10116 GN=rCG_34378 PE=3 SV=1	0	32	0	0.11	999999	999999
	A0A0G2KB77_RAT	4	60S ribosomal protein L17 OS=Rattus norvegicus OX=10116 PE=3 SV=1	0	32	0	0.14	999999	999999
	B2RZD1_RAT	4	Protein transport protein Sec61 subunit beta OS=Rattus norvegicus OX=10116 GN=Sec61b PE=1	0	32	0	0.33	999999	999999
	B5DEL9_RAT	4	40S ribosomal protein S7 OS=Rattus norvegicus OX=10116 GN=Rps7 PE=2 SV=1	0	31	0	0.31	999999	999999
	Q5BJN7_RAT	4	40S ribosomal protein S30 OS=Rattus norvegicus OX=10116 GN=LOC100360647 PE=1 SV=1	0	30	0	0.22	999999	999999
	D4A6G6_RAT	4	40S ribosomal protein S19 OS=Rattus norvegicus OX=10116 GN=LOC100362339 PE=3 SV=1	0	29	0	0.2	999999	999999
	H15_MOUSE	4	Histone H1.5 OS=Mus musculus OX=10090 GN=H1-5 PE=1 SV=2	0	29	0	0.14	999999	999999
	A0A0G2K719_RAT	4	RNA helicase OS=Rattus norvegicus OX=10116 GN=Ddx3x PE=1 SV=1	0	28	0	0.09	999999	999999
	A0A0U1RRV7_RAT	7	RCG61099, isoform CRA_b OS=Rattus norvegicus OX=10116 GN=Srsf3 PE=1 SV=1	0	28	0	0.23	999999	999999
	Q5FVQ2_RAT	4	Peter pan homolog OS=Rattus norvegicus OX=10116 GN=Ppan PE=1 SV=1	0	27	0	0.06	999999	999999
	D4ADF6_RAT	4	Zinc finger FYVE-type-containing 16 OS=Rattus norvegicus OX=10116 GN=Zfyve16 PE=1 SV=2	0	26	0	0.02	999999	999999
	M0RA26_RAT	4	Ribosomal protein S27-like OS=Rattus norvegicus OX=10116 GN=LOC100362987 PE=3 SV=1	0	25	0	0.35	999999	999999
	G3V798_RAT	4	Serine and arginine-rich-splicing factor 4 OS=Rattus norvegicus OX=10116 GN=Srsf4 PE=1 SV=1	0	25	0	0.06	999999	999999
	F1M3X8_RAT	7	Uncharacterized LOC102553785 OS=Rattus norvegicus OX=10116 GN=LOC102553785 PE=4 S\	0	25	0	0.05	999999	999999
	F1LX95_RAT	4	Dachshund family transcription factor 2 OS=Rattus norvegicus OX=10116 GN=Dach2 PE=4 SV=3	0	23	0	0.07	999999	999999
	A0A0G2K5X7_RAT	4	Histone H3 OS=Rattus norvegicus OX=10116 GN=RGD1564447 PE=3 SV=1	0	22	0	0.21	999999	999999
	A0A0G2JX92_RAT	7	SH3 and PX domains 2A OS=Rattus norvegicus OX=10116 GN=Sh3pxd2a PE=1 SV=1	0	22	0	0.03	999999	999999
	CYTSA_MOUSE	4	Cytospin-A OS=Mus musculus OX=10090 GN=Specc1l PE=1 SV=1	0	21	0	0.05	999999	999999
	E9PTB6_RAT	4	DNA-directed RNA polymerase subunit OS=Rattus norvegicus OX=10116 GN=Polr3a PE=3 SV=2	0	20	0	0.02	999999	999999
	Q6QI16_RAT	4	RNA transcription, translation and transport factor protein OS=Rattus norvegicus OX=10116 GN=R	0	20	0	0.09	999999	999999
	D4ADS6_RAT	4	Integrator complex subunit 7 OS=Rattus norvegicus OX=10116 GN=Ints7 PE=1 SV=1	0	20	0	0.03	999999	999999
	LTOR1_MOUSE	7	Ragulator complex protein LAMTOR1 OS=Mus musculus OX=10090 GN=Lamtor1 PE=1 SV=1	0	20	0	0.18	999999	999999
	A0A096MJS4_RAT	4	BRMS1-like transcriptional repressor (Fragment) OS=Rattus norvegicus OX=10116 GN=Brms1l P	0	19	0	0.12	999999	999999
	CHD1_MOUSE	4	Chromodomain-helicase-DNA-binding protein 1 OS=Mus musculus OX=10090 GN=Chd1 PE=1 S\	0	19	0	0.02	999999	999999
	CNTLN_MOUSE	4	Centlein OS=Mus musculus OX=10090 GN=Cntln PE=1 SV=1	0	19	0	0.02	999999	999999
	A0A0G2K506_RAT	6	Lactadherin OS=Rattus norvegicus OX=10116 GN=Mfge8 PE=1 SV=1	0	19	0	0.06	999999	999999
	A0A0G2JZI2_RAT	4	Glutamyl-tRNA synthetase OS=Rattus norvegicus OX=10116 GN=Eprs PE=1 SV=1	0	18	0	0.02	999999	999999
	EF1D_MOUSE	4	Elongation factor 1-delta OS=Mus musculus OX=10090 GN=Eef1d PE=1 SV=3	0	18	0	0.1	999999	999999
	SYFA_MOUSE	4	Phenylalanine--tRNA ligase alpha subunit OS=Mus musculus OX=10090 GN=Farsa PE=1 SV=1	0	18	0	0.05	999999	999999
	D3Z8W1_RAT	7	Uncharacterized protein OS=Rattus norvegicus OX=10116 PE=3 SV=1	0	18	0	0.22	999999	999999
	IF2A_MOUSE	4	Eukaryotic translation initiation factor 2 subunit 1 OS=Mus musculus OX=10090 GN=Eif2s1 PE=1 !	0	17	0	0.09	999999	999999
	SRF_MOUSE	4	Serum response factor OS=Mus musculus OX=10090 GN=Srf PE=1 SV=1	0	16	0	0.06	999999	999999
	A0A140TAB9_RAT	4	Protein-L-isoaspartate O-methyltransferase OS=Rattus norvegicus OX=10116 GN=Pcmt1 PE=1 S	0	16	0	0.15	999999	999999
	Q4V7B6_RAT	7	LOC499276 protein (Fragment) OS=Rattus norvegicus OX=10116 GN=LOC499276 PE=2 SV=1	0	16	0	0.08	999999	999999
	LAP2A_MOUSE	4	Lamina-associated polypeptide 2, isoforms alpha/zeta OS=Mus musculus OX=10090 GN=Tmpo P	0	15	0	0.04	999999	999999
	M0RB22_RAT	4	Protein-tyrosine-phosphatase OS=Rattus norvegicus OX=10116 GN=Ptprd PE=1 SV=2	0	15	0	0.01	999999	999999
	NOA1_MOUSE	7	Nitric oxide-associated protein 1 OS=Mus musculus OX=10090 GN=Noa1 PE=1 SV=1	0	15	0	0.04	999999	999999
	D3ZC46_RAT	4	Transcription factor 25 OS=Rattus norvegicus OX=10116 GN=Tcf25 PE=1 SV=2	0	14	0	0.04	999999	999999
	A0A0G2K283_RAT	4	RNA helicase OS=Rattus norvegicus OX=10116 GN=Dhx8 PE=1 SV=1	0	14	0	0.02	999999	999999

Supplementary Table S2 Primer information used in the study.

Primers for analysing the expression level of *Tmc1-8* in mouse DRG by qRT-PCR		
	Forward primer	Reverse primer
Tmc1	5’-ggatgttctgtcccaccctg-3’	5’-ggccttcacaagcttctcct-3’
Tmc2	5’-cagttaaagagcttggacgagg-3’	5’-atccttccggctagattggg-3’
Tmc3	5’-ggtagaggccctagccatcc-3’	5’-tggcagggaccttgtgattg-3’
Tmc4	5’-agatcacgtgtgggggaaac-3’	5’-aaactggaccaacagcgtga-3’
Tmc5	5’-tctctactggagtgaccgca-3’	5’-tgtttcggaccaagaggacg-3’
Tmc6	5’-gggccaccttcttccttacc-3’	5’-tcagccaacagctccttcag-3’
Tmc7	5’-gcagcgggccgtacac-3’	5’-tgccactgaccgatagcttg-3’
Tmc8	5’-ttgtgtttttctaaagcgtaagggc-3’	5’-catagccgctccacttcttg-3’
Primers for analysing the expression level of *Tmc6* in HEK293T cell and human DRG by qRT-PCR		
Tmc6	5’-tggccttcatcctcgatgt-3’	5’-tagtactgggagatgatggcac-3’
Primers and oligonucleotides for the construction of plasmids		
pCAG-IRES-*egfp*-m*Tmc6*	5’-cgagctcatggctcagtcactggctttag-3’	5’-ccggaattctcatggtgaccgggggttac-3’
p*egfp*-N3-2HA-m*Tmc6*	5’-<br>cgagctcatgtacccatatgatgtcccagactacgcatac<br>ccttatgacg<br>tgcctgactacgccatggctcagtcactggctttag-3’	5’-ccggaattctggtgaccgggggttac-3’
p*egfp*-N3-2HA-m*Tmc6*$^{\Delta 740-759}$	5’-ggagaggacaagatcttcctga-3’	5’-gatgttgaagtagatgacagcc-3’
p*egfp*-N3-2HA-m*Tmc6*$^{\Delta 760-779}$	5’-agcaggcctggcagaacccagg-3’	5’-ttcattccggatctgctccttg-3’
p*egfp*-N3-2HA-m*Tmc6*$^{\Delta 780-799}$	5’-gaccagaaggaaccctgtaacc-3’	5’-ccttccctcctcctcgtaaaca-3’
p*egfp*-N3-2HA-m*Tmc6*$^{\Delta 800-810}$	5’-tggaattctgcagtcgacggta-3’	5’-ccctccatcctcatgccaggca-3’
p*egfp*-N3-2HA-m*Tmc6*$^{\Delta 780-810}$	5’-tggaattctgcagtcgacggta-3’	5’-ccttccctcctcctcgtaaaca-3’
pCAG-IRES-*egfp*-*Gnaq*-3FLAG	5’-ctcaagcttcgaattatgactctggagtccatc-3’	5’-<br>gtcgactgcagaattttacttgtcatcgtcatccttgta<br>atcgatg<br>tcatgatctttataatcaccgtcatggtctttgtagtcg<br>accagattgtactcctt-3’
